# Diversity, morphology, and molecular phylogeny of *Diatrypaceae* from southern China

**DOI:** 10.3389/fmicb.2023.1140190

**Published:** 2023-04-06

**Authors:** Qi-Rui Li, Si-Han Long, Yan Lin, You-Peng Wu, Qian-Zhen Wu, Hong-Min Hu, Xiang-Chun Shen, Xu Zhang, Nalin Nilusha Wijayawardene, Ji-Chuan Kang, Jaturong Kumla, Ying-Qian Kang

**Affiliations:** ^1^State Key Laboratory of Functions and Applications of Medicinal Plants & Engineering and Research Center for Southwest Bio-Pharmaceutical Resources of National Education Ministry of China, Guizhou Medical University, Guiyang, China; ^2^The High Efficacy Application of Natural Medicinal Resources Engineering Center of Guizhou Province (The Key Laboratory of Optimal Utilization of Natural Medicine Resources), School of Pharmaceutical Sciences, Guizhou Medical University, Guiyang, China; ^3^Center for Yunnan Plateau Biological Resources Protection and Utilization, College of Biological Resource and Food Engineering, Qujing Normal University, Qujing, China; ^4^Section of Microbiology, Institute for Research and Development in Health and Social Care, Battaramulla, Sri Lanka; ^5^Engineering and Research Center for Southwest Bio-Pharmaceutical Resources of National Education Ministry of China, Guizhou University, Guiyang, China; ^6^Research Center of Microbial Diversity and Sustainable Utilization, Chiang Mai University, Chiang Mai, Thailand; ^7^Department of Biology, Faculty of Science, Chiang Mai University, Chiang Mai, Thailand; ^8^Key Laboratory of Environmental Pollution Monitoring and Disease Control, Ministry of Education, School of Basic Medical Sciences, Guizhou Medical University, Guiyang, China

**Keywords:** 8 new taxa, phylogeny, saprobe, taxonomy, *Xylariales*

## Abstract

During an investigation of *Diatrypaceae* from southern China, 10 xylariales-like taxa have been collected. Morphological and multi-gene analyses confirmed that these taxa reside in *Diatrypaceae* and represent eight novel taxa and two new records belonging to six genera *(viz*., *Allocryptovalsa, Diatrype, Diatrypella, Paraeutypella, Peroneutypa*, and *Vasilyeva* gen. nov.). *Vasilyeva* gen. nov. was proposed to accommodate *Vasilyeva cinnamomi* sp. nov. Among the other collections, seven new species were introduced (*viz*., *Diatrype camelliae-japonicae* sp. nov., *Diatrype rubi* sp. nov., *Diatrypella guiyangensis* sp. nov., *Diatrypella fatsiae-japonicae* sp. nov., *Paraeutypella subguizhouensis* sp. nov., *Peroneutypa hainanensis* sp. nov., and *Peroneutypa qianensis* sp. nov.), while two were reported as new records from China (*Allocryptovalsa rabenhorstii* and *Diatrype enteroxantha*). For *Diatrypaceae*, the traditional taxonomic approach based on morphology may not be applicable.

## Introduction

The family *Diatrypaceae* was erected by Nitschke (1870) to accommodate five genera *viz., Calosphaeria* Tul. & C. Tul., *Diatrype* Fr., *Diatrypella* (Ces. & De Not.) De Not., *Quaternaria* Tul. & C. Tul., and *Scoptria* Nitschke. The members of *Diatrypaceae* thrive in both aquatic and terrestrial habitats (Chlebicki, 1986; Glawe and Jacobs, 1987; Carmarán and Romero, 1992; Carmarán et al., 2006; Trouillas et al., 2010a; de Almeida et al., 2016), with different life modes, such as saprobes, pathogens, and endophytes, on economic crops and forest trees with a worldwide distribution (Vasilyeva and Ma, 2014; Dayarathne et al., 2016; Mayorquin et al., 2016; Senwanna et al., 2017; Hyde et al., 2020a; Konta et al., 2020). Phytopathogenic diatrypaceous taxa have been reported as causal agents of cankers, dieback, and grapevine trunk diseases (Glawe and Rogers, 1984; Rappaz, 1987; Trouillas and Gubler, 2004; Lardner et al., 2005; Luque et al., 2006; Catal et al., 2007), such as *Cryptosphaeria populina* (Pers.) Sacc., *Cryptosphaeria pullmanensis* Glawe, *Eutypa leptoplaca* (Durieu & Mont.) Rappaz, and *Eutypella parasitica* R.W. Davidson & R.C. Lorenz.

Kirk et al. (2008) accepted 13 genera in this family. Subsequently, *Allocryptovalsa* Senwanna et al., *Allodiatrype* Konta & K.D. Hyde, *Diatrypasimilis* Jian L. Zhou & Kohlm., *Halodiatrype* Dayar. & K.D. Hyde, *Halocryptosphaeria* Dayarath et al., *Halocryptovalsa* Dayar. & K.D. Hyde*, Monosporascus* Pollack & Uecker, *Neoeutypella* M. Raza et al., and *Pedumispora* K.D. Hyde & E.B.G. Jones were introduced as members of *Diatrypaceae* (Abdel-Wahab et al., 2014; Klaysuban et al., 2014; Maharachchikumbura et al., 2015; Dayarathne et al., 2016, 2020a,b; Senwanna et al., 2017; Phookamsak et al., 2019; Konta et al., 2020). In a recent study, Hyde et al. (2020b) and Wijayawardene et al. (2020) accepted 20 genera in *Diatrypaceae*. A total of 23 genera including five genera that lacks sequences data were accepted into the family by Zhu et al. (2021). Currently, 26 genera were included in *Diatrypaceae*, such as *Allocryptovalsa* Senwanna et al., *Allodiatrype* Konta & K.D. Hyde, *Anthostoma* Nitschke., *Cryptosphaeria* Ces. & De Not., *Cryptovalsa* Ces. & De Not., *Diatrypasimilis* Jian L. Zhou & Kohlm., *Diatrype* Fr., *Diatrypella* (Ces. & De Not.) De Not., *Dothideovalsa* Speg., *Echinomyces* Rappaz, *Endoxylina* Romell, *Eutypa* Tul. & C. Tul., *Eutypella* (Nitschke) Sacc., *Halocryptosphaeria* Dayarath., Devadatha, V.V. Sarma & K.D. Hyde, *Halocryptovalsa* Dayar. & K.D. Hyde, *Halodiatrype* Dayar. & K.D. Hyde, *Leptoperidia* Rappaz, *Libertella* Desm., *Monosporascus* Pollack & Uecker, *Neoeutypella* M. Raza, Q.J. Shang, Phookamsak & L. Cai, *Paraeutypella* L.S. Dissan., J.C. Kang, Wijayaw. & K.D. Hyde, *Pedumispora* K.D. Hyde & E.B.G. Jones, *Peroneutypa* Berl., *Pseudodiatrype* S.H. Long & Q.R. Li, *Quaternaria* Tul. & C. Tul., and *Rostronitschkia* Fitzp. (Hyde et al., 2020b; Konta et al., 2020; Dissanayake et al., 2021; Long et al., 2021; Samarakoon et al., 2022).

*Diatrypaceae* has been referred to as allantosporous taxa, which possess allantoid ascospores. Early classification systems of *Diatrypaceae* were mainly based on stromatal features including the degree of stromatal development, structure of perithecial necks, and type of host tissue (Fries, 1823; Glawe and Jacobs, 1987; Rappaz, 1987). Vasilyeva (1986) regarded that the morphology of the stromata causes significant confusion within *Diatrypaceae*.

A total of seven diatrypaceous species were known from the northeastern provinces of China before 2000 (Tai, 1979; Teng, 1996). Subsequent studies by Vasilyeva and Stephenson (2009) who carried out investigations in northeastern China, introduced nine species of pyrenomycetous fungi from China, including *Cryptosphaeria exornata, C. venusta*, and *Diatrype macounii*. In total, 15 species of *Diatrype, Diatrypella, Eutypa*, and *Eutypella* were documented by Vasilyeva from Heilongjiang province (Vasilyeva, 2011). A total of 13 species of *Diatrype* and *Cryptosphaeria* were collected from Heilongjiang and Jilin provinces by Vasilyeva and Ma (2014). Ma et al. (2016) reported *Cryptosphaeria pullmanensis* as the pathogens of a canker disease of willow and poplar in Xinjiang (*Paraeutypella* and a new species *Diatrypella longiasca* were reported from Guizhou by Dissanayake et al., 2021). In total, three new species (*Allodiatrype trigemina, Diatrype betulaceicola*, and *Diatrype larissae*) were reported based on morphological and molecular characteristics (Peng et al., 2021; Yang et al., 2022). Zhu et al. (2021) introduced nine novel species (*viz*. *Allocryptovalsa castaneae, A. castaneicola, Diatrype betulae, D. castaneicola, D. quercicola, Diatrypella betulae, D. betulicola, D. hubeiensis*, and *D. shennongensis*), a known species of *Diatrypella favacea* and a new host of *Eutypella citricola*, and asserted the high diversity of *Diatrypaceae* in China. Long et al. (2021) made a new contribution to *Diatrypaceae* from karst areas in China and figured out that the number of ascospores per ascus is not a good diagnostic feature at the genus level.

During the investigation of *Xylariales* from south China, 20 samples belonging to 12 species of *Diatrypaceae* were collected. Based on morpho-molecular analyses, a new genus (*viz. Vasilyeva*), eight new species, and two new country records are reported in this study.

## Materials and methods

### Collection, morphology, and isolation

During the rainy seasons of 2020–2021, 20 samples of *Diatrypaceae* on dead woods and barks were collected from south China (Guizhou, Hainan, and Yunnan Provinces). The samples were stored in paper bags and taken back to the lab for examination. Macro-morphological characteristics were examined and photographed using a camera fixed to the Olympus SZ61 stereo microscope (Olympus Corporation, Japan). Microscopic examinations were carried out using a Nikon Ni compound microscope (Nikon Corporation, Japan), and photographs were taken using a Canon 550 camera. More than 30 asci and ascospores were measured with Tarosoft (R) Image Frame Work (v.0.9.7). Graphic plates were arranged with Adobe Photoshop v. CS6.

Single-spore isolation was obtained following the method of Chomnunti et al. (2014). The ascospores were picked into a small amount of sterile water, mixed well, and smeared on a potato dextrose agar (PDA). After 12 h, the germination of ascospores was observed using a stereomicroscope, and the germinated ascospores were transferred to a new PDA plate in a sterile environment. The specimens were deposited at the Herbarium of Cryptogams, Herbarium of Kunming Institute of Botany, Chinese Academy of Sciences (KUN-HKAS), Yunnan province, China, and Herbarium of Guizhou Medical University (GMB), Guizhou Province, China. The cultures were deposited at the Guizhou Medical University Culture Collection (GMBC). Nomenclatural novelties were deposited in the MycoBank (Crous et al., 2004).

### DNA extraction, PCR amplification, and sequencing

Colonies were grown on PDA for ~1 week until the hyphae covered the plate. Mycelium was scraped off using a sterile scalpel for DNA extraction. Total DNA was extracted from fresh mycelia using the BIOMIGA Fungus Genomic DNA Extraction Kit following its instruction. The segments of the internal transcribed spacer (ITS) region, large-subunit (LSU) ribosomal RNA, β-tubulin (*tub2*), and RNA polymerase II subunit (*rpb2*) genes were amplified separately by primer pairs ITS4/ITS5, LR0R/LR5, and T1/T22 (T1/Bt2b and Bt2a/Bt2b), and RPB2-5f/RPB2-7Cr, respectively (Vilgalys and Hester, 1990; White et al., 1990; Glass and Donaldson, 1995; O'Donnell and Cigelnik, 1997). The PCR amplification conditions were performed following the study of Long et al. (2021). PCR products were checked with the gel electrophoresis method and sent to Sangon Biotech (Shanghai) Co., Ltd. for sequencing. All new sequences were uploaded on GenBank (https://www.ncbi.nlm.nih.gov/).

### Sequence alignment and phylogenetic analyses

Sequences for alignment were downloaded from GenBank and are presented in Table 1. The sequences mainly referred to recent articles, such as Zhu et al. (2021) and Long et al. (2021). The dataset of combined ITS and β-tubulin gene alignments was aligned using MAFFT (http://mafft.cbrc.jp/alignment/server/index.html) (Katoh and Standley, 2013). Multi-gene sequence alignment was assembled using BioEdit 7.2.6.1. Phylip file for RAxML analyses and Nexus file for Bayesian analyses were obtained on the phylogeny website tools ALTER (http://sing.ei.uvigo.es/ALTER/) (Glez-Peña et al., 2010).

**Table 1:** Isolates and GenBank accession numbers used in the phylogenetic analyses of *Diatrypaceae*.

**Species**	**Strain number**	**GenBank Accession number**		**References**
		**ITS**	β**-tubulin**	
*Allocryptovalsa castaneae*	CFCC52428^T^	MW632945	MW656393	Zhu et al., 2021
*Allocryptovalsa castaneicola*	CFCC52432^T^	MW632947	MW656395	Zhu et al., 2021
*Allocryptovalsa cryptovalsoidea*	HVFIG02^T^	HQ692573	HQ692524	Trouillas et al., 2011
*Allocryptovalsa elaeidis*	MFLUCC 15-0707^T^	MN308410	MN340296	Konta et al., 2020
*Allocryptovalsa polyspora*	MFLUCC 17–0364^T^	MF959500	MG334556	Senwanna et al., 2017
*Allocryptovalsa rabenhorstii*	WA08CB	HQ692619	HQ692523	Trouillas et al., 2011
***Allocryptovalsa rabenhorstii***	**GMB0416**	**OP935171**	**OP938733**	**This study**
*Allocryptovalsa sichuanensis*	HKAS 107017^T^	MW240633	MW775592	Samarakoon et al., 2022
*Allocryptovalsa xishuangbanica*	KUMCC 21-0830^T^	ON041128	ON081498	Maharachchikumbura et al., 2022
***Allocryptovalsa xishuangbanica***	**GMB0417**	**OP935176**	**OP938739**	**This study**
*Allodiatrype albelloscutata*	IFRD 9100 ^T^	OK257020	NA	Li et al., 2022
*Allodiatrype arengae*	MFLUCC 15-0713^T^	MN308411	MN340297	Konta et al., 2020
*Allodiatrype elaeidicola*	MFLUCC 15-0737a^T^	MN308415	MN340299	Konta et al., 2020
*Allodiatrype elaeidis*	MFLUCC 15-0708a^T^	MN308412	MN340298	Konta et al., 2020
*Allodiatrype taiyangheensis*	IFRDCC2800^T^	OK257021	OK345036	Li et al., 2022
*Allodiatrype thailandica*	MFLUCC 15-3662	KU315392	NA	Li et al., 2016
*Allodiatrype trigemina*	FCATAS 842 ^T^	MW031919	MW371289	Peng et al., 2021
*Alloeutypa flavovirens*	E48C, CBS 272.87	AJ302457	DQ006959	Rolshausen et al., 2006
*Alloeutypa milinensis*	FCATAS4309^T^	OP538689	OP557595	Ma et al., 2023
*Alloeutypa milinensis*	FCATAS4382^T^	OP538690	OP557596	Ma et al., 2023
*Anthostoma decipiens*	IPV-FW349	AM399021	AM920693	Unpublished
*Anthostoma decipiens*	JL567	JN975370	JN975407	Luque et al., 2012
*Cryptosphaeria eunomia*	C1C, CBS 216.87	AJ302417	NA	Acero et al., 2004
*Cryptosphaeria eunomia*	C5C, CBS 223.8	AJ302421	NA	Acero et al., 2004
*Cryptosphaeria ligniota*	CBS 273.87	KT425233	KT425168	Acero et al., 2004
*Cryptosphaeria pullmanensis*	ATCC 52655	KT425235	KT425170	Trouillas et al., 2015
*Cryptosphaeria subcutanea*	DSUB100A	KT425189	KT425124	Trouillas et al., 2015
*Cryptosphaeria subcutanea*	CBS 240.87	KT425232	KT425167	Trouillas et al., 2015
*Cryptovalsa ampelina*	A001	GQ293901	GQ293972	Trouillas et al., 2010b
*Cryptovalsa ampelina*	DRO101	GQ293902	GQ293982	Trouillas et al., 2010b
*Cryptovalsa elevata*	CBS 125574	MH863711	NA	Vu et al., 2019
*Diatrype betulaceicola*	FCATAS 2725^T^	OM040386	OM240966	Yang et al., 2022
*Diatrype betulae*	CFCC52416 ^T^	MW632943	MW656391	Zhu et al., 2021
***Diatrype betulae***	**GMB0426**	**OP935181**	**OP938750**	**This study**
*Diatrype bullata*	UCDDCh400	DQ006946	DQ007002	Rolshausen et al., 2006
***Diatrype camelliae-japonicae***	**GMB0427** ^**T**^	**OP935172**	**OP938734**	**This study**
***Diatrype camelliae-japonicae***	**GMB0428**	**OP935173**	**OP938735**	**This study**
*Diatrype castaneicola*	CFCC52425^T^	MW632941	MW656389	Zhu et al., 2021
*Diatrype disciformis*	GNA14	KR605644	KY352434	Senanayake et al., 2015
*Diatrype disciformis*	D21C, CBS 205.87	AJ302437	NA	Acero et al., 2004
*Diatrype enteroxantha*	HUEFS155114	KM396617	KT003700	de Almeida et al., 2016
*Diatrype enteroxantha*	HUEFS155116	KM396618	KT022236	de Almeida et al., 2016
***Diatrype enteroxantha***	**GMB0433**	**OP935170**	**OP938736**	**This study**
*Diatrype lancangensis*	GMB0045^T^	MW797113	MW814885	Long et al., 2021
*Diatrype lancangensis*	GMB0046	MW797114	MW814886	Long et al., 2021
*Diatrype larissae*	FCATAS 2723^T^	OM040384	OM240964	Yang et al., 2022
*Diatrype lijiangensis*	MFLU 19-0717^T^	MK852582	MK852583	Thiyagaraja et al., 2019
*Diatrype palmicola*	MFLUCC 11-0020^T^	KP744438	NA	Liu et al., 2015
*Diatrype palmicola*	MFLUCC 11-0018	KP744439	NA	Liu et al., 2015
*Diatrype quercicola*	CFCC52418^T^	MW632938	MW656386	Zhu et al., 2021
***Diatrype rubi***	**GMB0429** ^**T**^	**OP935182**	**OP938740**	**This study**
***Diatrype rubi***	**GMB0430**	**OP935183**	**OP938741**	**This study**
*Diatrype spilomea*	D17C	AJ302433	NA	Acero et al., 2004
*Diatrype stigma*	DCASH200	GQ293947	GQ294003	Trouillas et al., 2010b
*Diatrype undulata*	D20C, CBS 271.87	AJ302436	NA	Acero et al., 2004
*Diatrypella atlantica*	HUEFS 136873	KM396614	KR259647	de Almeida et al., 2016
*Diatrypella betulae*	CFCC52406^T^	MW632931	MW656379	Zhu et al., 2021
*Diatrypella betulicola*	CFCC52411^T^	MW632935	MW656383	Zhu et al., 2021
*Diatrypella banksiae*	CPC 29118	KY173402	NA	Crous et al., 2016
*Diatrypella delonicis*	MFLUCC 15-1014	MH812994	MH847790	Hyde et al., 2019
*Diatrypella delonicis*	MFLU 16-1032	MH812995	MH847791	Hyde et al., 2019
*Diatrypella elaeidis*	MFLUCC 15-0279	MN308417	MN340300	Konta et al., 2020
***Diatrypella fatsiae-japonica***	**GMB0422** ^**T**^	**OP935184**	**OP938744**	**This study**
***Diatrypella fatsiae-japonicae***	**GMB0423**	**OP935185**	**OP938745**	**This study**
*Diatrypella favacea*	Islotate 380	KU320616	NA	de Almeida et al., 2016
*Diatrypella favacea*	DL26C	AJ302440	NA	Unpublished
*Diatrypella frostii*	UFMGCB 1917	HQ377280	NA	Vieira et al., 2011
***Diatrypella guiyangensis***	**GMB0414** ^**T**^	**OP935188**	**OP938742**	**This study**
***Diatrypella guiyangensis***	**GMB0415**	**OP935189**	**OP938743**	**This study**
*Diatrypella heveae*	MFLUCC 15-0274	MN308418	MN340301	Konta et al., 2020
*Diatrypella heveae*	MFLUCC 17-0368^T^	MF959501	MG334557	Senwanna et al., 2017
*Diatrypella hubeiensis*	CFCC 52413^T^	MW632937	NA	Zhu et al., 2021
*Diatrypella iranensis*	KDQ18^T^	KM245033	KY352429	Mehrabi et al., 2015
*Diatrypella longiasca*	KUMCC 20-0021^T^	MW036141	MW239658	Dissanayake et al., 2021
*Diatrypella macrospora*	KDQ15^T^	KR605648	KY352430	Mehrabi et al., 2016
*Diatrypella oregonensis (Diatrype oregonensis*	DPL200	GQ293940	GQ293999	Trouillas et al., 2010b
*Diatrypella oregonensis (Diatrype oregonensis*	CA117	GQ293934	GQ293996	Trouillas et al., 2010b
*Diatrypella pseudooregonensis*	GMB0039^T^	MW797115	MW814888	Long et al., 2021
*Diatrypella pseudooregonensis*	GMB0040	MW797117	MW814889	Long et al., 2021
*Diatrypella pulvinata*	H048	FR715523	FR715495	de Almeida et al., 2016
*Diatrypella pulvinata*	DL29C	AJ302443	NA	Unpublished
*Diatrypella tectonae*	MFLUCC 12-0172a^T^	KY283084	NA	Shang et al., 2017
*Diatrypella tectonae*	MFLUCC 12-0172b^T^	KY283085	KY421043	Shang et al., 2017
*Diatrypella verruciformis*	UCROK1467	JX144793	JX174093	Lynch et al., 2013
*Diatrypella verruciformis*	UCROK754	JX144783	JX174083	Lynch et al., 2013
*Diatrypella vulgaris*	HVFRA02	HQ692591	HQ692503	Trouillas et al., 2011
*Diatrypella vulgaris*	HVGRF03	HQ692590	HQ692502	Trouillas et al., 2011
*Diatrypella yunnanensis*	VT01^T^	MN653008	MN887112	Zhu et al., 2021
*Eutypa armeniacae*	ATCC 28120	DQ006948	DQ006975	Rolshausen et al., 2006
*Eutypa astroidea*	E49C, CBS 292.87	AJ302458	DQ006966	Rolshausen et al., 2006
*Eutypa camelliae*	HKAS 107022^T^	MW240634	MW775593	Samarakoon et al., 2022
*Eutypa cerasi*	GMB0048^T^	MW797104	MW814893	Long et al., 2021
*Eutypa cerasi*	GMB0049	MW797105	MW814877	Long et al., 2021
*Eutypa laevata*	E40C CBS 291.87	AJ302449	NA	Acero et al., 2004
*Eutypa lata*	CBS290.87	HM164736	HM164770	Trouillas and Gubler, 2010
*Eutypa lata*	EP18	HQ692611	HQ692501	Trouillas et al., 2011
*Eutypa lata*	RGA01	HQ692614	HQ692497	Trouillas et al., 2011
*Eutypa lejoplaca*	CBS 248.87	DQ006922	DQ006974	Rolshausen et al., 2006
*Eutypa leptoplaca*	CBS 287.87	DQ006924	DQ006961	Rolshausen et al., 2006
*Eutypa maura*	CBS 219.87	DQ006926	DQ006967	Rolshausen et al., 2006
*Eutypa microasca*	BAFC 51550	KF964566	KF964572	Grassi et al., 2014
*Eutypa sparsa*	3802 3b	AY684220	AY684201	Trouillas and Gubler, 2004
*Eutypa tetragona*	CBS 284.87	DQ006923	DQ006960	Rolshausen et al., 2006
*Eutypella caricae*	EL51C	AJ302460	NA	Acero et al., 2004
*Eutypella cearensis*	HUEFS 131070^T^	KM396639	NA	de Almeida et al., 2016
*Eutypella cerviculata*	M68^T^	JF340269	NA	Arhipova et al., 2012
*Eutypella cerviculata*	EL59C^T^	AJ302468	NA	Acero et al., 2004
*Eutypella leprosa*	EL54C	AJ302463	NA	Acero et al., 2004
*Eutypella leprosa*	Isolate 60	KU320622	NA	de Almeida et al., 2016
*Eutypella microtheca*	BCMX01	KC405563	KC405560	Paolinelli-Alfonso et al., 2015
*Eutypella motuoensis*	FCATAS4035^T^	OP538695	NA	Ma et al., 2023
*Eutypella motuoensis*	FCATAS4082^T^	OP538693	OP557599	Ma et al., 2023
*Eutypella parasitica*	CBS 210.39	DQ118966	NA	Jurc et al., 2006
*Eutypella quercina*	IRANC2543C ^T^	KX828139	KY352449	Mehrabi et al., 2019
*Eutypella semicircularis*	MP4669	JQ517314	NA	Mehrabi et al., 2016
*Eutypella tamaricis*	MFLUCC 14-0444	KU900330	KX453302	Thambugala et al., 2017
*Halocryptovalsa salicorniae*	MFLUCC 15-0185	MH304410	MH370274	Dayarathne et al., 2020b
*Halodiatrype avicenniae*	MFLUCC 15-0953	KX573916	KX573931	Dayarathne et al., 2016
*Halodiatrype salinicola*	MFLUCC 15-1277^T^	KX573915	KX573932	Dayarathne et al., 2016
*Kretzschmaria deusta*	CBS 826.72	KU683767	KU684190	U'ren et al., 2016
*Monosporascus cannonballus*	CMM3646^T^	JX971617	NA	Unpublished
*Monosporascus cannonballus*	ATCC 26931^T^	FJ430598	NA	Unpublished
*Neoeutypella baoshanensis*	LC 12111^T^	MH822887	MH822888	Hyde et al., 2019
*Neoeutypella baoshanensis*	EL51C, CBS 274.87^T^	AJ302460	NA	Acero et al., 2004
*Paraeutypella citricola*	HVVIT07	HQ692579	HQ692512	Trouillas et al., 2011
*Paraeutypella citricola*	HVGRF01	HQ692589	HQ692521	Trouillas et al., 2011
*Paraeutypella guizhouensis*	KUMCC 20-0016^T^	MW039349	MW239660	Dissanayake et al., 2021
*Paraeutypella guizhouensis*	KUMCC 20-0017	MW036141	MW239661	Dissanayake et al., 2021
*Paraeutypella pseudoguizhouensis*	**GMB0420** ^**T**^	**OP935186**	**OP938748**	**This study**
*Paraeutypella pseudoguizhouensis*	**GMB0421**	**OP935187**	**OP938749**	**This study**
*Paraeutypella vitis*	UCD2291AR	HQ288224	HQ288303	Úrbez-Torres et al., 2012
*Paraeutypella vitis*	UCD2428TX	FJ790851	GU294726	Úrbez-Torres et al., 2009
*Pedumispora rhizophorae*	BCC44877^T^	KJ888853	NA	Klaysuban et al., 2014
*Pedumispora rhizophorae*	BCC44878^T^	KJ888854	NA	Klaysuban et al., 2014
*Peroneutypa alsophila*	EL58C, CBS 250.87	AJ302467	NA	Acero et al., 2004
*Peroneutypa curvispora*	HUEFS 136877^T^	KM396641	NA	de Almeida et al., 2016
*Peroneutypa diminutiasca*	MFLUCC 17-2144^T^	MG873479	NA	Shang et al., 2018
*Peroneutypa diminutispora*	HUEFS 192196 ^T^	KM396647	NA	de Almeida et al., 2016
*Peroneutypa hainanensis*	**GMB0424** ^**T**^	**OP935179**	**OP938746**	**This study**
*Peroneutypa hainanensis*	**GMB0425**	**OP935180**	**OP938747**	**This study**
*Peroneutypa indica*	NFCCI 4393^T^	MN061368	MN431498	Dayarathne et al., 2020a
*Peroneutypa kochiana*	EL53M	AJ302462	NA	Carmarán et al., 2006
*Peroneutypa kunmingensis*	HKAS 113189^T^	MZ475070	MZ490589	Phukhamsakda et al., 2022
*Peroneutypa leucaenae*	MFLU 18-0816^T^	MW240631	MW775591	Samarakoon et al., 2022
*Peroneutypa longiasca*	MFLU 17-1217^T^	MF959502	MG334558	Senwanna et al., 2017
*Peroneutypa mackenziei*	MFLUCC 16-0072^T^	KY283083	KY706363	Shang et al., 2017
*Peroneutypa mangrovei*	PUFD526^T^	MG844286	MH094409	Phookamsak et al., 2019
*Peroneutypa qianensis*	**GMB0431** ^**T**^	**OP935177**	**NA**	**This study**
*Peroneutypa qianensis*	**GMB0432**	**OP935178**	**NA**	**This study**
*Peroneutypa polysporae*	NFCCI 4392^T^	MN061367	MN431497	Dayarathne et al., 2020a
*Peroneutypa rubiformis*	MFLU 17-1185 ^T^	MG873477	MH316763	Shang et al., 2018
*Pseudodiatrype hainanensis*	GMB0054^T^	MW797111	MW814883	Long et al., 2021
*Pseudodiatrype hainanensis*	GMB0055	MW797112	MW814884	Long et al., 2021
*Quaternaria quaternata*	EL60C, CBS 278.87	AJ302469	NA	Acero et al., 2004
*Quaternaria quaternata*	GNF13	KR605645	NA	Mehrabi et al., 2016
***Vasilyeva cinnamomi***	**GMB0418** ^**T**^	**OP935174**	**OP938737**	**This study**
***Vasilyeva cinnamomi***	**GMB0419**	**OP935175**	**OP938738**	**This study**
*Xylaria hypoxylon*	CBS 122620	AM993141	KX271279	Peršoh et al., 2009

T indicates type strain. NA, No sequence is available in GenBank; Newly generated sequences are indicated in bold. ATCC, American Type Culture Collection; CBS, Westerdijk Fungal Biodiversity Institute (CBS-KNAW Fungal Biodiversity Centre), Utrecht, The Netherlands; CFCC, China Forestry Culture Collection Center; GMB, Herbarium of Guizhou Medical University, China; HKAS, Cryptogams Herbarium of Kunming Institute of Botany Academia Sinica; HUEFS, Herbarium of the State University of Feira de Santana; MFLUCC, Mae Fah Luang University Culture Collection, Thailand; Others, information not available.

Maximum likelihood (ML) analyses were carried out on the CIPRES Science Gateway v.3.3 (http://www.phylo.org/portal2; Miller et al., 2010), using RAxML v.8.2.8 as of the ‘RAxML-HPC BlackBox' tool (Stamatakis and Ott, 2008). GTRGAMMA + I model was selected. The best-scoring tree was selected with a final ML optimization likelihood value of −20426.053370. Branch support (BS) for ML analyses was calculated by 1,000 bootstrap replicates.

The best-fit evolution model for each dataset for Bayesian inference (BI) was calculated with MrModeltest 2.3. The GTR+I+G model of DNA substitution and a gamma distribution rate variation across sites were selected for the construction of a Bayesian phylogenetic tree (Ronquist and Huelsenbeck, 2003). Posterior probabilities (PPs) (Rannala and Yang, 1996) were determined by Markov Chain Monte Carlo sampling (MCMC) (Ronquist and Huelsenbeck, 2003). A total of six simultaneous Markov chains were run from random starting trees for 1.2 million generations, and trees were sampled every 1,000 generations. The first 25% of generations were discarded as burn-in. The remaining trees were used to calculate the posterior probabilities in the majority rule consensus tree. Phylogenetic trees were visualized with FigTree v.1.4.4 and annotated by software of Microsoft Office PowerPoint and Adobe Photoshop v. CS6.

## Results

### Phylogenetic analyses

**Figure 1:**
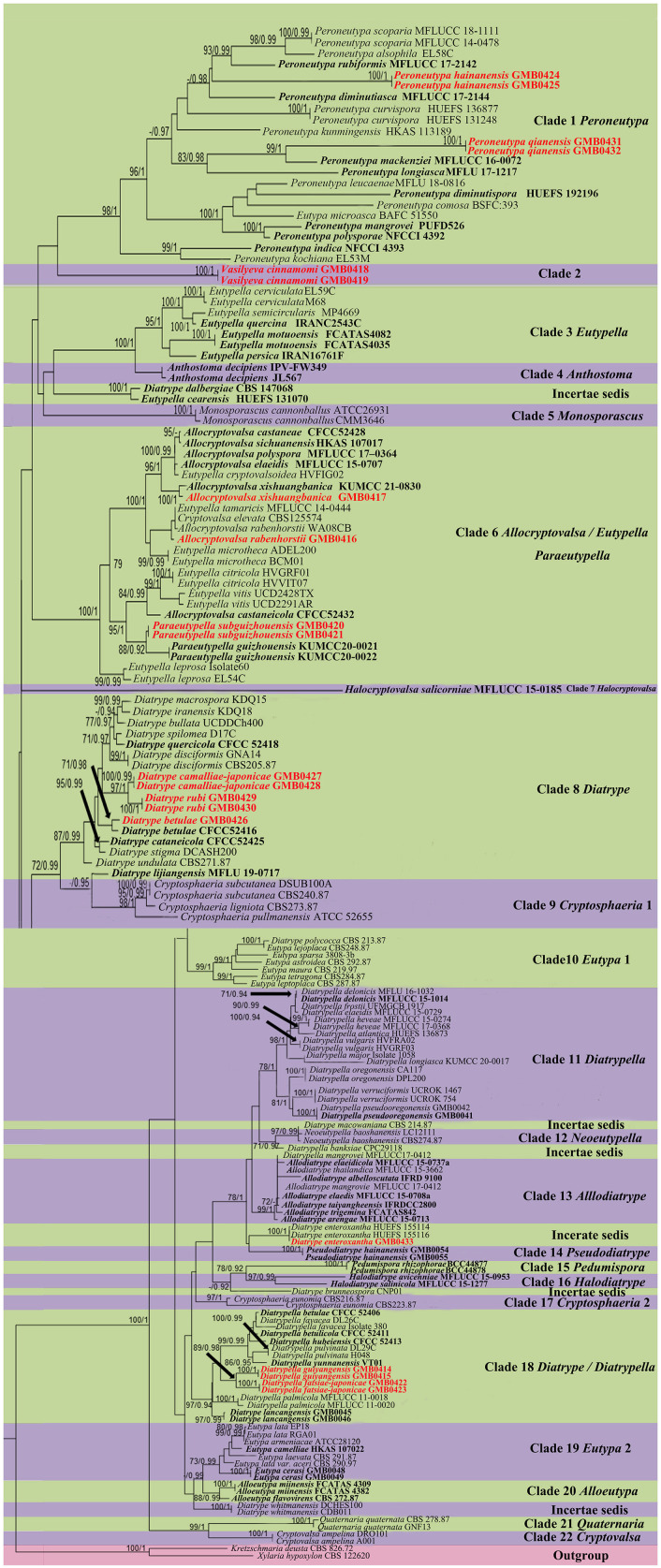
Phylogram generated from maximum likelihood (RAxML) analyses, based on ITS-β-tubulin matrix. ML bootstrap supports (≥70%) and Bayesian posterior probability (≥0.90) are indicated as ML/BYPP. The tree is rooted to *Kretzschmaria deusta* (CBS 826.72) and *Xylaria hypoxylon* (CBS 122620). Ex-type strains are in black bold. Newly generated strains are in red bold.

The topologies of RAxML and BYPP analyses were similar to overall tree topologies and did not differ significantly. The dataset consists of 171 taxa for representative strains of species in *Diatrypaceae*, including outgroup taxa with 1,451 characteristics and gaps (ITS: 1–487 and β-tubulin: 488–1451). The RAxML analyses resulted in a best-scoring likelihood tree which is shown in Figure 1.

The phylogenetic tree contains 22 clades within *Diatrypaceae*. *Peroneutypa hainanensis* and *Peroneutypa qianensis* cluster with *Peroneutypa species* in Clade 1, *Peroneutypa hainanensis* formed a distinct branch basal to *Peroneutypa alsophila, P. rubiformis, and P. scoparia*, and *P. qianensis* was sister to *P. mackenziei* with the high bootstrap support (99/1). *Vasilyeva* formed a separate branch sister to *Peroneutypa* with low bootstrap support (42/0.84). In clade 6, *Paraeutypella subguizhouensis* formed a sister clade to *Paraeutypella guizhouensis* with moderate bootstrap and PP support, respectively (88/0.92). This clade is not well-resolved and comprises three genera *viz. Allocryptovalsa, Eutypella*, and *Paraeutypella*. In clade 8, *Diatrype camelliae-japonicae* and *D. rubi* formed a distinct branch in clade 8 and clustered with *Diatrype s. str*., and *D. betulae* (GMB0426) formed a sister clade with ex-type strain *Diatrype betulae* CFCC52416 with high bootstrap support (71/0.98). *Diatrype camelliae-japonicae* and *D. rubi* were introduced as two new species. *Diatrype betulae* (GMB0426) was introduced with the sexual morph. *Diatrypella guiyangensis* and *D. fatsiae-japonicae* formed a separate branch in clade 18, which is an unsolved clade that contains *Diatrype* and *Diatrypella*.

### Taxonomy

A total of 12 taxa of *Diatrypaceae* were collected from southern China, including one new genus, eight new species, two new records for China, and two known species.

***Allocryptovalsa*** Senwanna, Phookamsak & K.D. Hyde, Mycosphere 8(10): 1839 (2017).

**MycoBank No:** MB 553857.

**Notes**: The genus *Allocryptovalsa* was introduced by Senwanna et al. (2017) and was typified with *A. polyspora* C. Senwanna et al. This genus was characterized by present or absent stromata mostly in the bark, asci clavate to spindle-shape, long pedicellate, polysporous asci, and allantoid to sub-allantoid ascospores (Senwanna et al., 2017). The asexual morph was not determined. In this study, we report a new record of *Allocryptovalsa rabenhorstii* and re-describe a known species of *Allocryptovalsa xishuangbanica* from China.

**Figure 2:**
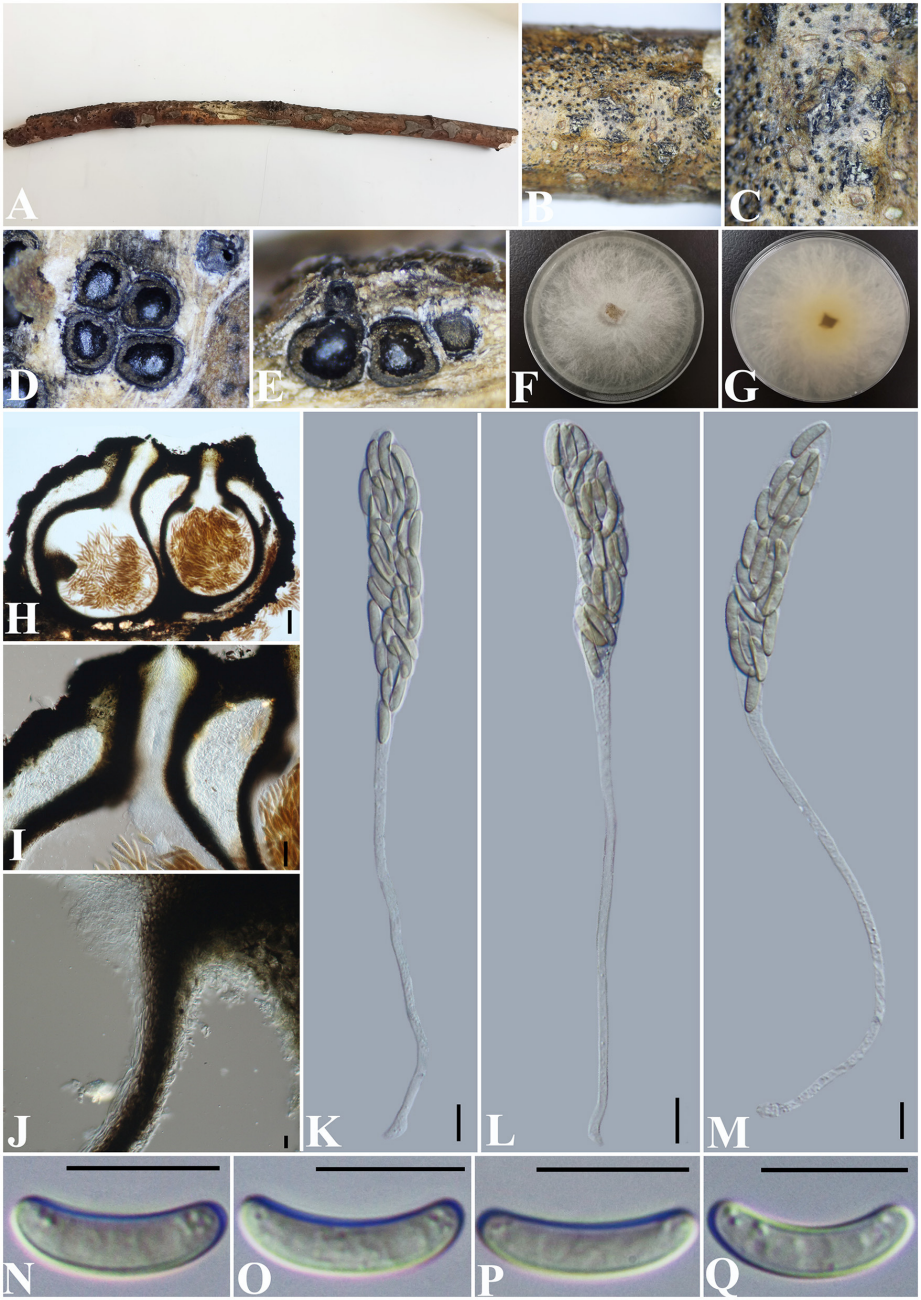
***Allocryptovalsa rabenhorstii*** (GMB0416). **(A)** Material. **(B, C)** Close-up of stromata. **(D)** Transverse section through stromata. **(E)** Vertical section through stromata. **(F, G)** Culture on PDA. **(H)** Section through the ascostroma. **(I)** Ostiolar canal. **(J)** Peridium. **(K–M)** Asci. **(N–Q)** Ascospores. Scale bars: **(H)** = 100 μm; **(I)** = 50 μm; **(J–Q)** = 10 μm.

***Allocryptovalsa rabenhorstii*** (Nitschke) C. Senwanna, Phookamsak & K.D. Hyde, Mycosphere 8(10): 1841 (2017) (Figure 2).

**Basionym:** *Valsa rabenhorstii* Nitschke, Pyrenomyc. Germ. 1: 158.

**MycoBank No:** MB 553864.

**Material examined:** China, Guizhou Province, Qianxinan Buyi Miao Autonomous Prefecture, Anlong County (25°5′53.44″N, 105°26′33.64″E) on branches of an unidentified plant, 24 September 2021, Altitude: 833 m, S. H. Long & Q. R. Li., ALX4-2 (GMB0416, **new record from China**), living culture GMBC0416.

*Saprobic* on a dead twig of an unidentified plant. **Sexual morph**: ***Stromata*** solitary to gregarious, 1–4 loculate, immersed to semi-immersed, becoming raised to erumpent through the bark. ***Perithecia*** 380–550 μm diameter, 625–800 μm high, globose to subglobose, dark brown to black, ostiolate, papillate, perithecial, dark brown to black, gregarious or solitary, immersed to semi-immersed in the substrate. ***Ostioles*** opening separately, papillate, central. ***Peridium*** 35–50 μm wide, composed of two types of layers of cells, the outer layer comprising several layers of thick-walled, dark brown to black textura angularis cells, the inner layer comprising 3–5 layers of thin-walled, hyaline textura angularis cells. ***Asci*** 170– 230 × 11.5–20 μm ($\bar{\text{x}}$ = 202.8 × 15.4 μm, *n* = 30), polysporous, unitunicate, thin-walled, clavate, long pedicellate, apically rounded. ***Ascospores*** 12.5–17.5 × 3–4 μm ($\bar{\text{x}}$ = 14.8 × 3.4 μm, *n* = 30), crowded, pale yellowish to pale brown at maturity, oblong to allantoid, aseptate, slightly curved, smooth-walled, with small guttules. **Asexual morph**: Undetermined.

**Culture characteristics**: Ascospores germinating on PDA within 24 h. Colonies on PDA, white when young, became light yellow, dense, but thinning toward the edge, margin rough, white from above, reverse white at margin, light yellow at the center, no pigmentation, and no sporulation produced on the PDA medium.

**Notes:** In morphology, our new collection of *Allocryptovalsa rabenhorstii* (GMB0416) resembles *Allocryptovalsa s.str*. Sequences generated from the cultures of *Allocryptovalsa rabenhorstii* (GMB0416) are similar to *Allocryptovalsa rabenhorstii* WA08CB (ITS: 99.1%, 3/434 gaps; BT: 99.0%, 0/200 gaps). *Allocryptovalsa rabenhorstii* has been previously reported from Australia and Iran (Trouillas et al., 2011; Mehrabi et al., 2016), and this is the first report of *Allocryptovalsa rabenhorstii* from China.

**Figure 3:**
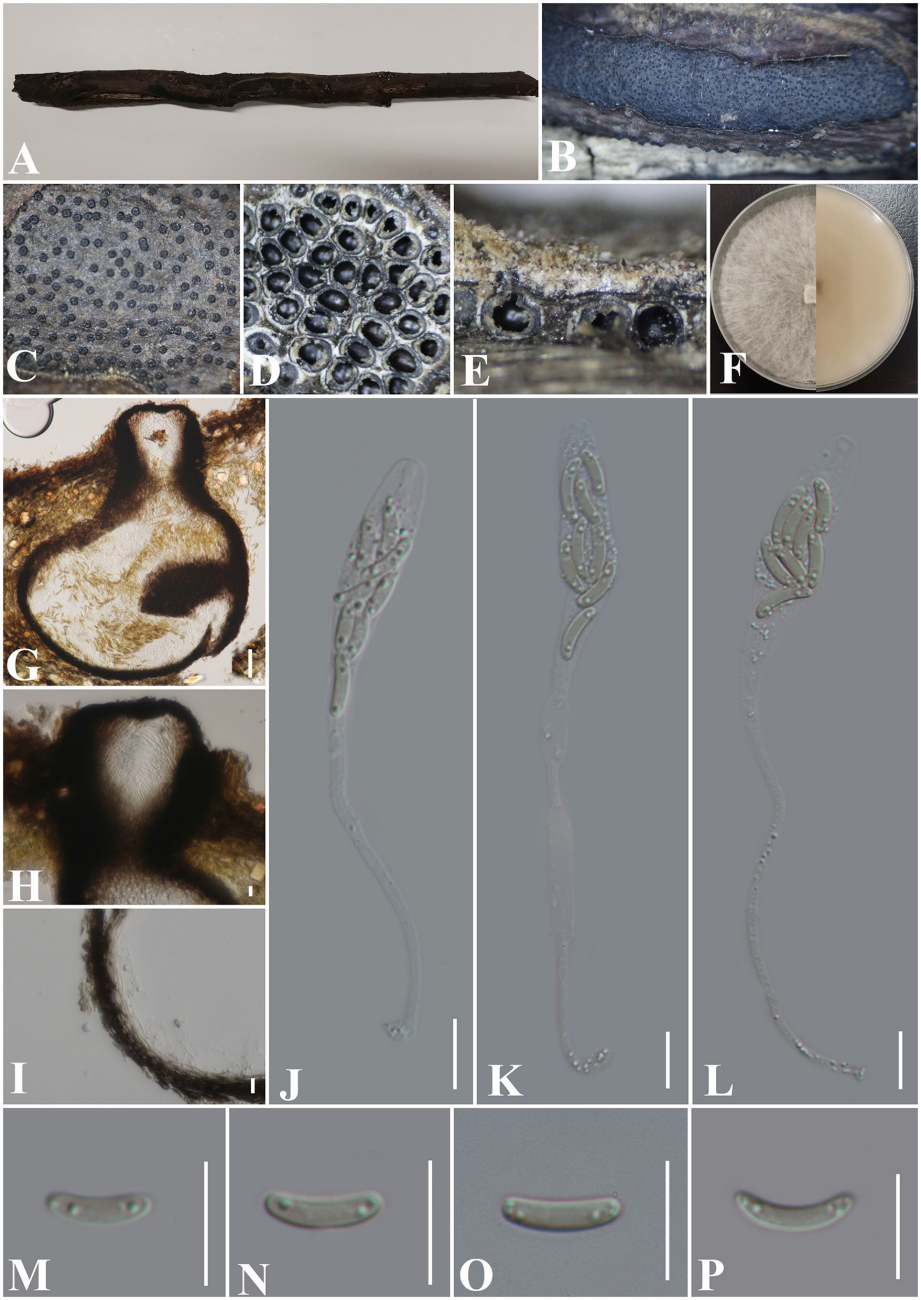
***Allocryptovalsa xishuangbanica*** (GMB0417). **(A)** Material. **(B, C)** Close-up of stromata. **(D)** Transverse section through stromata. **(E)** Vertical section through stromata. **(F)** Cultures on PDA. **(G)** Section through the ascostroma. **(H)** Ostiolar canal. **(I)** Peridium. **(J–L)** Asci. **(M–P)** Ascospores. Scale bars: **(G)** = 50 μm; **(H–P)** = 10 μm.

***Allocryptovalsa xishuangbanica*** Maharachch. & Wanas., Life 12(5, no. 635): 9 (2022) (Figure 3).

**MycoBank No**: MB 843438.

**Material examined**: China, Guizhou Province, Anshun City, Pingba District (26°20'36.23“N, 106°19'20.68”E) on branches of *Bombax ceiba* Linnaeus, 12 December 2021, Altitude: 1220 m, S. H. Long & Q. R. Li., PB200 (GMB0417, first **report from Guizhou Province, China**), living culture GMBC0417.

*Saprobic* on the surface of *Bombax ceiba* branches. **Sexual morph:** ***Stromata*** 1.5–4.5 cm long and 0.3–0.5 cm broad ($\bar{\text{x}}$ = 2.6 × 0.4 cm, *n* = 30), ~0.4 mm high, well-developed, erumpent through the bark, irregular in shape, widely effused, flat, margin diffuse, surface dark brown to black, with punctiform ostioles scattered at the surface. Regions between perithecia necks are occupied by white pseudoparenchymatous entostromatic tissue. ***Endostroma*** consists of an outer layer of black, small, dense, and thin parenchymal cells and an inner layer of white, large, and loose parenchymal cells. ***Perithecia*** 200–324 μm high, 346–477 μm diameter ($\bar{\text{x}}$. =250 × 408 μm, *n* = 10), immersed in stromata, globose to subglobose with ostiole, the tissue between perithecia is white. ***Ostioles*** opening separately, papillate, central. ***Peridium*** 30–50 μm thick, dark brown to hyaline with textura angularis cell layers. ***Asci*** 81.5–142 × 5–11 μm ($\bar{\text{x}}$ = 120.7 × 9.0 μm, *n* = 30), 8-spored, unitunicate, long-cylindrical, with long stipe, rounded to truncate apex, apical rings inamyloid. ***Ascospores*** 8–12 × 1.8–3 μm ($\bar{\text{x}}$ = 10 × 2.4 μm, *n* = 30), overlapping, allantoid, slightly curved, subhyaline, smooth, aseptate, usually with small guttules at ends. **Asexual morph**: Undetermined.

**Culture characteristics**: Ascospores germinating on PDA within 24 h. Colonies on PDA, white when young, became luteous, dense, but thinning toward the edge, margin rough, white from above, reverse white to luteous, no pigmentation, and no sporulation produced on the PDA medium.

**Notes:** Figure 1 shows that our new collection (GMB0417) belongs to the genus *Allocryptovalsa*. Morphologically, GMB0417 closely resembles *Allocryptovalsa xishuangbanica* (HKAS122936, holotype), such as immersed or semi-immersed stromata, but GMB0417 has longer asci (81.5–142 × 5–11 μm vs. 60–80 × 7–10 μm) and slightly longer ascospores (8–12 × 1.8–3 μm vs. 7–10.5 × 1.8–2.6 μm) (Maharachchikumbura et al., 2022). The ITS sequence of *Allocryptovalsa xishuangbanica* GMB0417 is similar to the ITS sequence of *A. xishuangbanica* (HKAS122936) (99.2%, 0/476 gaps). Based on the molecular data, we identified it as *Allocryptovalsa xishuangbanica*. This species was originally introduced from the Yunnan province, China, but this is the first report from the Guizhou province, China.

***Diatrype*** Fr.

**MycoBank No**: MB 1504.

**Notes**: The genus *Diatrype* was introduced by Fries (1849) with *Diatrype disciformis* as the generic type. The genus is characterized by stromata widely effuse or verrucose, flat or slightly convex, with discoid or sulcate ostioles at the surface, 8-spored and long-stalked asci, and hyaline or brownish, allantoid ascospores. The asexual morph of *Diatrype* is reported as libertella-like and dumortieria-like (Kirk et al., 2008; Maharachchikumbura et al., 2015; Senanayake et al., 2015). In this study, we introduce two new species (*viz*., *Diatrype camelliae-japonicae and Diatrype rubi*) while reporting a new record of *Diatrype enteroxantha* and a known species of *Diatrype betulae* from China.

**Figure 4:**
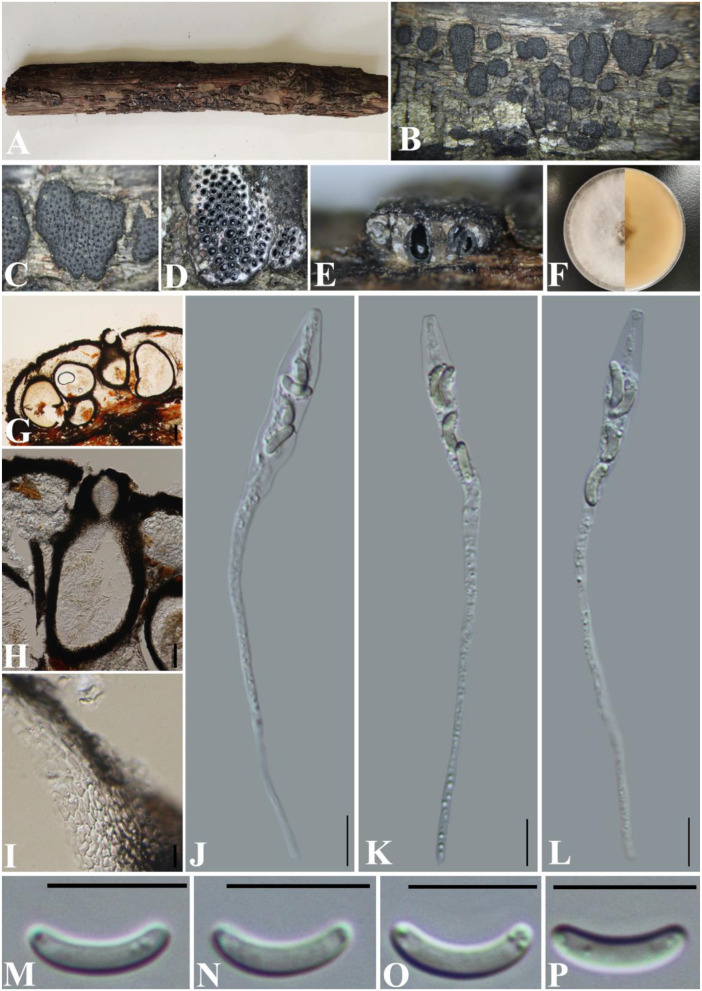
***Diatyrpe betulae*** (GMB0426). **(A)** Material. **(B, C)** Close-up of stromata. **(D)** Transverse section through stromata. **(E)** Vertical section through stromata. **(F)** Culture on PDA. **(G)** Section through the ascostroma. **(H)** Ostiolar canal. **(I)** Peridium. **(J–L)** Asci. **(M–P)** Ascospores. Scale bars: **(G)** = 50 μm; **(H–P)** = 10 μm.

***Diatrype betulae*** H.Y. Zhu & X.L. Fan, Frontiers in Microbiology 12(no. 646262): 8 (2021) (Figure 4).

**MycoBank No**: MB 837784.

**Material examined**: China, Yunnan Province, Chuxiong Yi Autonomous Prefecture, Chuxiong city, Zixi Mountain (25°1′15.13″N, 107°23′48.44″E) on branches of an unidentified plant, 2 August 2021, Altitude: 2314 m, S. H. Long & Q. R. Li., ZXS04 (GMB0426, first **report of sexual morph**), living culture GMBC0426.

*Saprobic* on the surface of dead wood. **Sexual morph:** ***Stromata*** 1.4–3.3 mm diameter, ~0.5–0.7 mm thick, erumpent through the bark, extending into a black area, aggregated, circular to irregular in shape, flat, margin diffused, surface dark brown to black, with punctiform ostioles scattered on the surface, with tissues soft, white between perithecia. ***Entostroma*** dark with embedded perithecia in one layer. ***Perithecia*** 370–580 μm high, 200–270 μm broad ($\bar{\text{x}}$ = 415.5 × 248.0 μm, *n* = 10), semi-immersed in stromata, globose to subglobose, glabrous, with a short neck. ***Ostioles*** opening separately, papillate, central. ***Peridium*** 25–40 μm thick, dark brown to hyaline with *textura angularis* cell layers. ***Asci*** 77–122 × 5.5–8.5 μm ($\bar{\text{x}}$ = 106 × 6.8 μm *n* = 30), 8-spored, unitunicate, long-cylindrical, with long stipe, rounded, apical rings inamyloid. ***Ascospores*** 8.5–12 × 1.5–2.5 μm ($\bar{\text{x}}$ = 10.1 × 1.7 μm, *n* = 30), overlapping, allantoid, curved, hyaline, smooth, aseptate, usually with small guttules. **Asexual morph**: See Zhu et al. (2021).

**Culture characteristics**: Ascospores germinating on PDA within 24 h. Colonies on PDA, white when young, became light brown, dense, but thinning toward the edge, margin rough, white from above, reverse white at the margin, mauve to sepia and at the center, no pigmentation, and no sporulation produced on the PDA medium.

**Notes:** *Diatrype betulae* (CFCC 52416, ex-type) was introduced by Zhu et al. (2021) only based on the asexual morph. In the phylogenetic analyses, our new collection (GMB0426) formed a sister clade with *Diatrype betulae* CFCC 52416 with moderate bootstrap and PP support, respectively (71/0.98). ITS sequence of GMB0426 is similar to that generated from *Diatrype betulae* (CFCC 52416, ex-type) (ITS: 99.6%, 0/479 gaps). Based on the phylogenetic analyses and megablast, we conclude GMB0426 is representing the sexual morph of *Diatrype betulae*, and this is the first time reporting its sexual morph.

**Figure 5:**
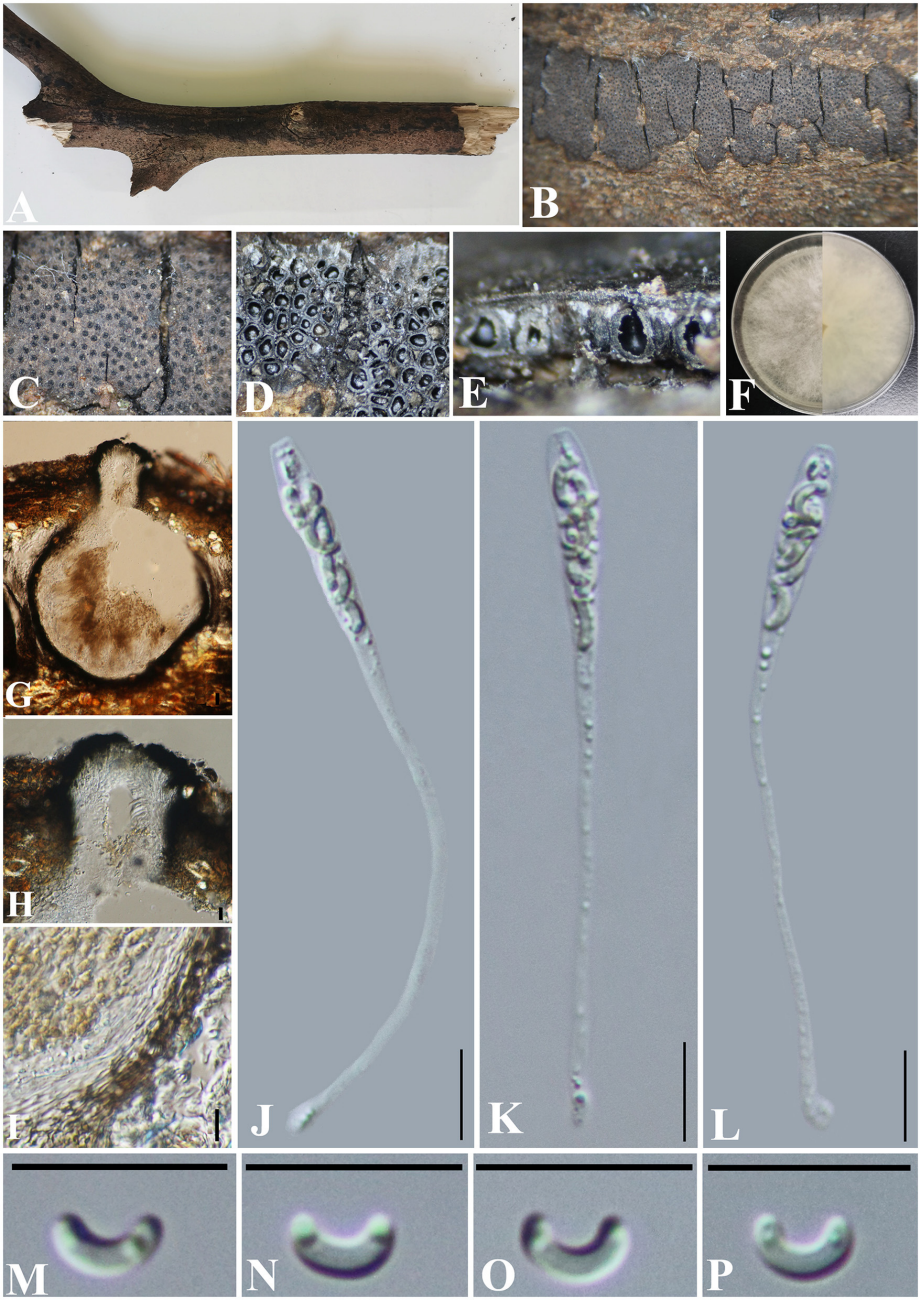
***Diatrype camelliae-japonicae*** (GMB0427, holotype). **(A)** Type material. **(B, C)** Close-up of stromata. **(D)** Transverse section through stromata. **(E)** Vertical section through stromata. **(F)** Culture on PDA. **(G)** Section through the ascostroma. **(H)** Ostiolar canal. **(I)** Peridium. **(J–L)** Asci. **(M–P)** Ascospores. Scale bars: **(G)** = 50 μm; **(H–P)** = 10 μm.

***Diatrype camelliae-japonicae*** S. H. Long & Q. R. Li. sp. nov. (Figure 5).

**MycoBank No**: MB 846768.

**Etymology**: Refers to its host, *Camellia japonica* L.

**Material examined**: China, Guizhou Province, Qiannan Buyi Miao Autonomous Prefecture, Duyun City, Doupeng Mountain (26°21′49.23″N, 107°22′36.25″E) on branches of *Camellia japonica* L., 7 July 2021, Altitude: 1105 m, S. H. Long & Q. R. Li., DPS20 (GMB0427, **holotype**), ex-type GMBC0427; *ibid* (KUN-HKAS 126458, **isotype**).

*Saprobic* on branches of *Camellia japonica*. **Sexual morph**: ***Stromata*** 0.2–6 cm long and 0.4–1 cm board, ~0.5 mm thick, erumpent through the bark, extending into a black area, aggregated, irregular in shape, widely effused, flat, margin diffused, surface dark brown to black, with punctiform ostioles scattered at the surface, with tissues soft, white between perithecia. ***Entostroma*** dark with embedded perithecia in one layer. ***Perithecia*** 230–380 μm high, 170–220 μm broad ($\bar{\text{x}}$ = 315.5 × 198.0 μm, *n* = 10), semi-immersed in the stroma, globose to subglobose, glabrous, with cylindrical neck. ***Ostioles*** opening separately, papillate or apapillate, central. ***Peridium*** 25–40 μm thick, dark brown to hyaline with textura angularis cell layers. ***Asci*** 74–107 × 3.8–5.5 μm ($\bar{\text{x}}$ = 85.5 × 4.7 μm *n* = 30), 8-spored, unitunicate, long-cylindrical, with long stipe, rounded, apical rings inamyloid. ***Ascospores*** 5.0–7.6 × 1.2–2.8 μm ($\bar{\text{x}}$ = 6.6 × 1.4 μm, *n* = 30), overlapping, allantoid, curved, hyaline, smooth, aseptate, usually with small guttules. **Asexual morph**: Undetermined.

**Culture characteristics**: Ascospores germinating on PDA within 24 h. Colonies on PDA, white when young, became light brown, dense, but thinning toward the edge, margin rough, white from above, reverse white at the margin, mauve to sepia and at the center, no pigmentation, and no sporulation produced on the PDA medium.

**Additional material examined:** China, Guizhou Province, Qiannan Buyi Miao Autonomous Prefecture, Duyun City, Doupeng Mountain (26°21′30.19″N, 107°22′9.55″E) on branches of an unidentified plant, 7 July 2021, Altitude: 1292 m, S. H. Long & Q. R. Li., DPS183 (GMB0428, **paratype**, ex-paratype GMBC0428).

**Notes:** In the phylogenetic analyses, *Diatrype camelliae-japonicae* formed a distinct clade in *Diatrype*. Morphologically, the stromata of *Diatrype camelliae-japonicae* are similar to *D. stigma, D. undulata, D. hypoxyloides, D. playstoma*, and *D. subundulata* (Vasilyeva and Ma, 2014). However, the ascospores of GMB0027 are shorter than those of *D. playstoma* (7–9 × 1–1.3 μm) and *D. subundulata* (7–9 × 1.7–1.9 μm) and wider than those of *D. undulata* (5–7 × 0.9–1.3 μm) and *D. hypoxyloides* (4–6 μm long, very thin) (Vasilyeva and Ma, 2014). Moreover, the ascospores of *D. camelliae-japonicae* are hyaline while *D. subundulata* and *D. undulate* have yellowish ascospores (Vasilyeva and Ma, 2014). *Diatrype camelliae-japonicae* can be distinguished from *D. stigma* since the ascospores of the former are moderately curved, while those of the latter are straight (Vasilyeva and Ma, 2014).

**Figure 6:**
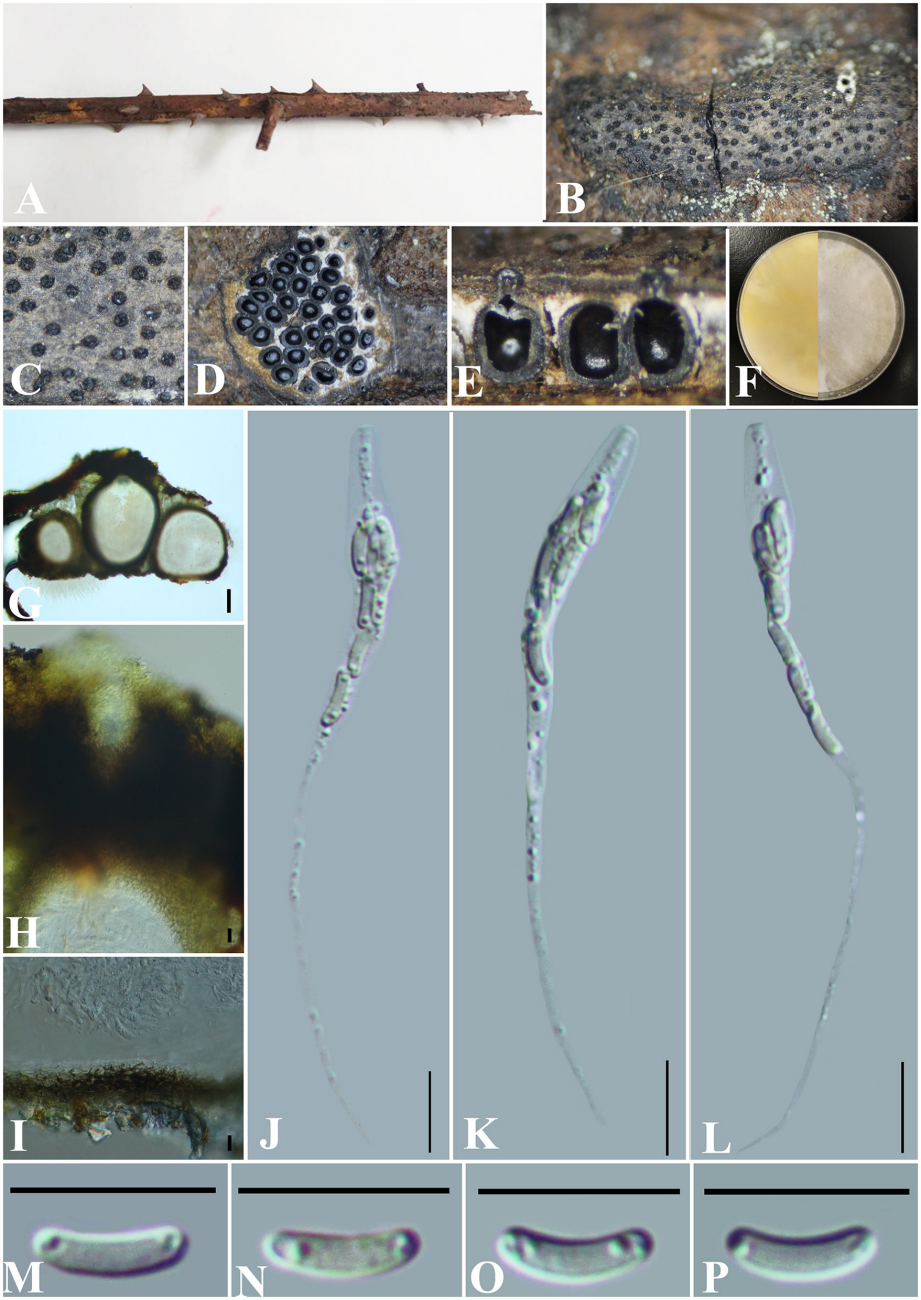
***Diatrype rubi*** (GMB0429, holotype). **(A)** Type material. **(B, C)** Close-up of stromata. **(D)** Transverse section through stromata. **(E)** Vertical section through stromata. **(F)** Culture on PDA. **(G)** Section through the ascostroma. **(H)** Ostiolar canal. **(I)** Peridium. **(J–L)** Asci. **(M–P)** Ascospores. Scale bars: **(G)** = 50 μm; **(H–P)** = 10 μm.

***Diatrype rubi*** S. H. Long & Q. R. Li. sp. nov. (Figure 6).

**MycoBank No**: MB 846769.

**Etymology**: Refers to its host, *Rubus corchorifolius* L. f.

**Material examined**: China, Guizhou Province, Qiannan Buyi Miao Autonomous Prefecture, Dushan County, Jingshin Valley Scenic Area (25°82′49.23″N, 107°54′36.25″E) on branches of *Rubus corchorifolius* L. f., 18 November 2021, Altitude: 1001 m, S. H. Long & Q. R. Li., JXG3 (GMB0429, **holotype**), ex-type GMBC0429; *ibid* (KUN-HKAS 126459, **isotype**).

*Saprobic* on the branch surface of *Rubus corchorifolius*. **Sexual morph:** ***Stromata*** 0.2–0.7 cm long and 0.15–0.4 cm broad ($\bar{\text{x}}$ = 0.4 × 0.25 mm, *n* = 30), ~0.5 mm thick, semi-immersed through host bark, irregular in shape, widely effused, margin diffused, surface dark brown, with punctiform ostioles scattered at the surface, with tissues soft, white between perithecia. ***Entostroma*** dark with embedded perithecia in one layer. ***Perithecia*** semi-immersed in the stroma, globose to subglobose, glabrous, with cylindrical neck, brevicollous or longicollous, 287–500 μm high, 200–294 μm broad ($\bar{\text{x}}$ = 369.5 × 245.5 μm, *n* = 10), ovoid, obovoid to oblong, monostichous, aterrimus. ***Ostioles*** opening separately, papillate or apapillate, central. ***Peridium*** 20–30 μm thick, dark brown to hyaline with textura angularis cell layers. ***Asci*** 73–97 × 4–6 μm ($\bar{\text{x}}$ = 79 × 5.2 μm *n* = 30), 8-spored, unitunicate, long-cylindrical, with long stipe, rounded, apical rings inamyloid. ***Ascospores*** 6.5–8 × 1.5–2 μm ($\bar{\text{x}}$ = 6.9 × 1.5 μm, *n* = 30), overlapping, allantoid, straight to slightly curved, hyaline, smooth, aseptate, usually with small guttules. **Asexual morph**: Undetermined.

**Culture characteristics**: Ascospores germinating on PDA within 24 h. Colonies on PDA, white when young, became light yellow, dense but thinning toward the edge, margin rough, white from above, reverse white at the margin, mauve to sepia and at the center, no pigmentation, and no sporulation produced on the PDA medium.

**Additional material examined**: China, Guizhou Province, Qiannan Buyi Miao Autonomous Prefecture, Dushan County, Jingshin Valley Scenic Area (25°82′70.33″N, 107°54′31.23″E) on branches of thorns, 18 November 2021, Altitude: 1,001 m, S. H. Long & Q. R. Li., JXG11 (GMB0430, **paratype**, ex-paratype GMBC0430).

**Notes:** Phylogenetic analyses show that *Diatrype rubi* has a close relationship with *D. camelliae-japonicae* (Figure 1). Morphologically, the stromata of *D. rubi* is similar to *D. stigma, D. undulata, D. hypoxyloides, D. playstoma*, and *D. subundulata*, but the ascospores of *D. rubi* are wider than those of *D. playstoma* (7–9 × 1–1.3 μm) (Vasilyeva and Ma, 2014). The ascospores of *D. undulata* (5–7 × 0.9–1.3 μm) and *D. hypoxyloides* (4–6 long, very thin) are narrower than those of *D. rubi* (Vasilyeva and Ma, 2014). The ascospores of *D. rubi* are hyaline while *D. subundulata* and *D. undulate* have yellowish ascospores (Vasilyeva and Ma, 2014). In addition, *Diatrype rubi* can be distinguished from *D. stigma* by its longer asci (73–97 × 4–6 μm vs. 25–30 × 5–7 μm) (Vasilyeva and Ma, 2014) and from *D. camelliae-japonicae* by the size of ascospores (6.5–8 × 1.5–2 μm vs. 5.0–7.6 × 1.2–2.8). Moreover, the ascospores of *D. rubi* are straight to slightly curved, and the ascospores of *D. camelliae-japonicae* are slightly curved. Here, we introduce *Diatrype rubi* based on both morpho-molecular analyses.

**Figure 7:**
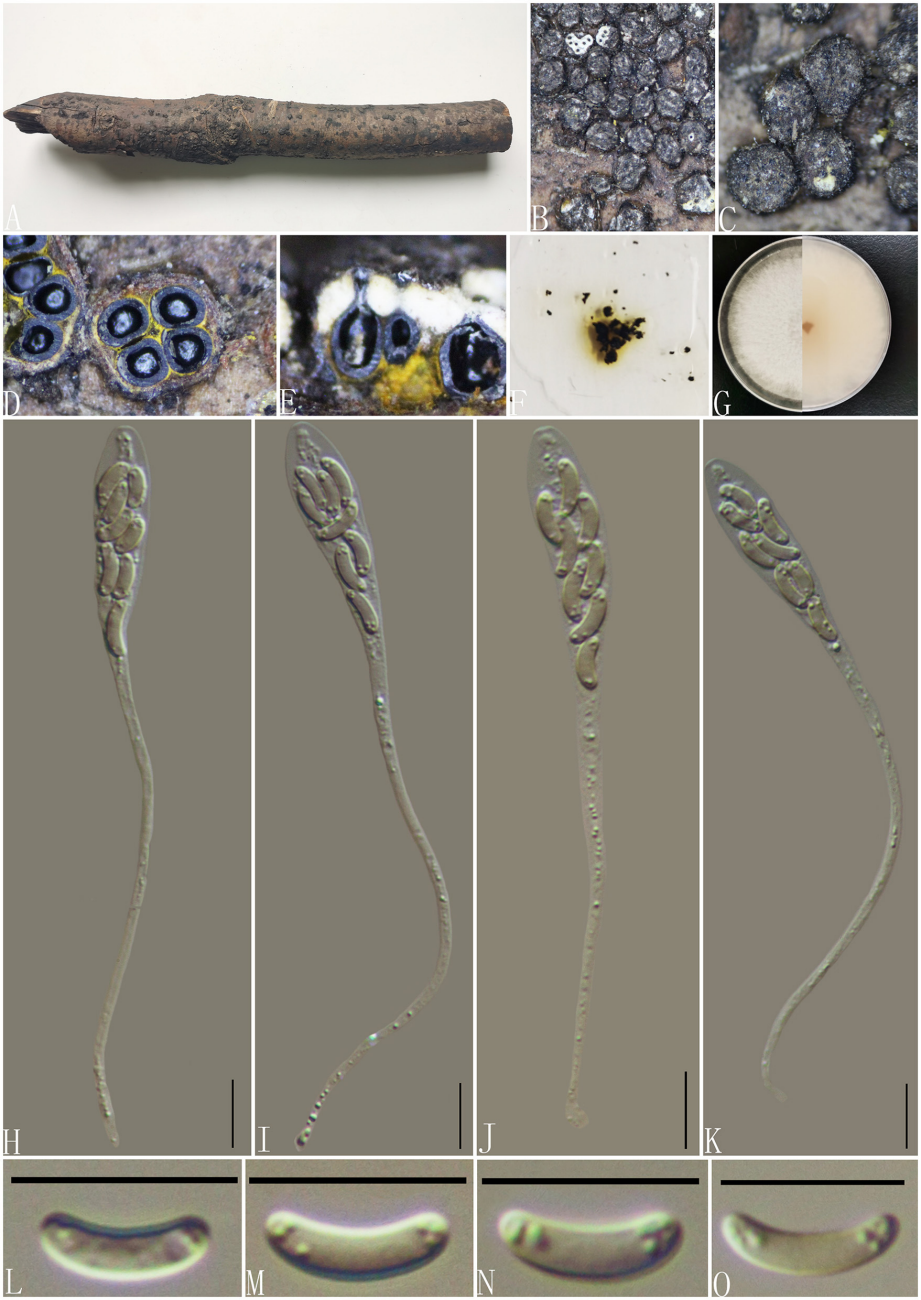
***Diatrype enteroxantha*** (GMB0433). **(A)** Material. **(B, C)** Close-up of stromata. **(D)** Transverse section through stromata. **(E)** Vertical section through stromata. **(F)** Pigments in KOH. **(G)** Culture on PDA. **(H–K)** Asci. **(L–O)** Ascospores. Scale bars: **(H–O)** = 10 μm.

***Diatrype enteroxantha*** (Sacc.) Berl., Icon. fung. (Abellini) 3(3-4): 93 (1902) (Figure 7).

**MycoBank No**: MB 454899.

**Material examined**: China, Guizhou Province, Guiyang City, Huaxi Wetland Park (26°11′33.23″N, 106°54′10.11″E) on branches of an unidentified plant, 7 October 2020, Altitude: 1,140 m, S. H. Long & Q. R. Li., HX10 (GMB0433, **new record from China**), living culture GMBC0433.

*Saprobic* on the surface of dead wood. **Sexual morph**: ***Stromata*** 0.9–2.55 mm in diameter, 0.6–1 mm high, erumpent through the bark, irregular to circular in shape, solitary to gregarious, and surface dark brown to black. ***Entostroma*** **is** composed of two parts; the base region was bases occupied by thin, powdery, yellow tissue, and the entostromatic region between perithecial necks occupied by thick, white tissue. ***Perithecia*** 520–640 μm high, 230–260 μm broad ($\bar{\text{x}}$ = 315.5 × 198 μm, *n* = 10), globose to subglobose, glabrous, with cylindrical neck. ***Ostioles*** opening separately, papillate or apapillate, central. ***Peridium*** 30–40 μm thick, dark brown to hyaline with *textura angularis* cell layers. ***Asci*** 94–133 × 7–9.5 μm ($\bar{\text{x}}$ = 117.2 × 8.4 μm *n* = 30), 8-spored, unitunicate, long-cylindrical, with long stipe, apically rounded to truncate, apical rings inamyloid. ***Ascospores*** 7–10.5 × 1.5–2.5 μm ($\bar{\text{x}}$ = 8.5 × 2 μm, *n* = 30), overlapping, allantoid, slightly curved, hyaline, smooth, aseptate, usually with small guttules. **Asexual morph**: Not formed.

**Culture characteristics:** Ascospores germinating on PDA within 24 h. Colonies on PDA, white when young, became light brown, dense, but thinning toward the edge, margin rough, white from above, reverse white at the margin, mauve to sepia and at the center, no pigmentation, and no sporulation produced on the PDA medium.

**Notes:** In the phylogenetic analyses, GMB0433 clusters with the strains *Diatrype enteroxantha* HUEFS 155114 and HUEFS 155116 with a high support value (100/1) (Figure 1). Morphologically, GMB0433 is consistent with the descriptions of the holotype of *D. enteroxantha* (Rappaz, 1987). Sequences of GMB0433 are similar to *Diatrype enteroxantha* (HUEFS 155116) (ITS: 99.4%, 3/501 gaps). *Diatrype enteroxantha* has been reported in Argentina, Brazil, Guyana, and South Africa (Doidge, 1941; Rappaz, 1987; de Almeida et al., 2016), and this is the first report from Asia and China.

***Diatrypella*** (Ces. & De Not.) De Not.

**MycoBank No**: MB 1505.

**Note**: The genus *Diatrypella* was introduced by Cesati and De Notaris (1863) and was typified with *Diatrypella verruciformis* (Ehrh.) Nitschke. This genus was characterized by pustule-like stromata erumpent through the host surface, polysporous asci and allantoid ascospores, and libertella-like asexual morphs (Senanayake et al., 2015; Hyde et al., 2017; Shang et al., 2017). In this study, we introduced two new species of *Diatrypella* (*viz., Diatrypella fatsiae-japonica, Diatrypella guiyangensis*).

**Figure 8:**
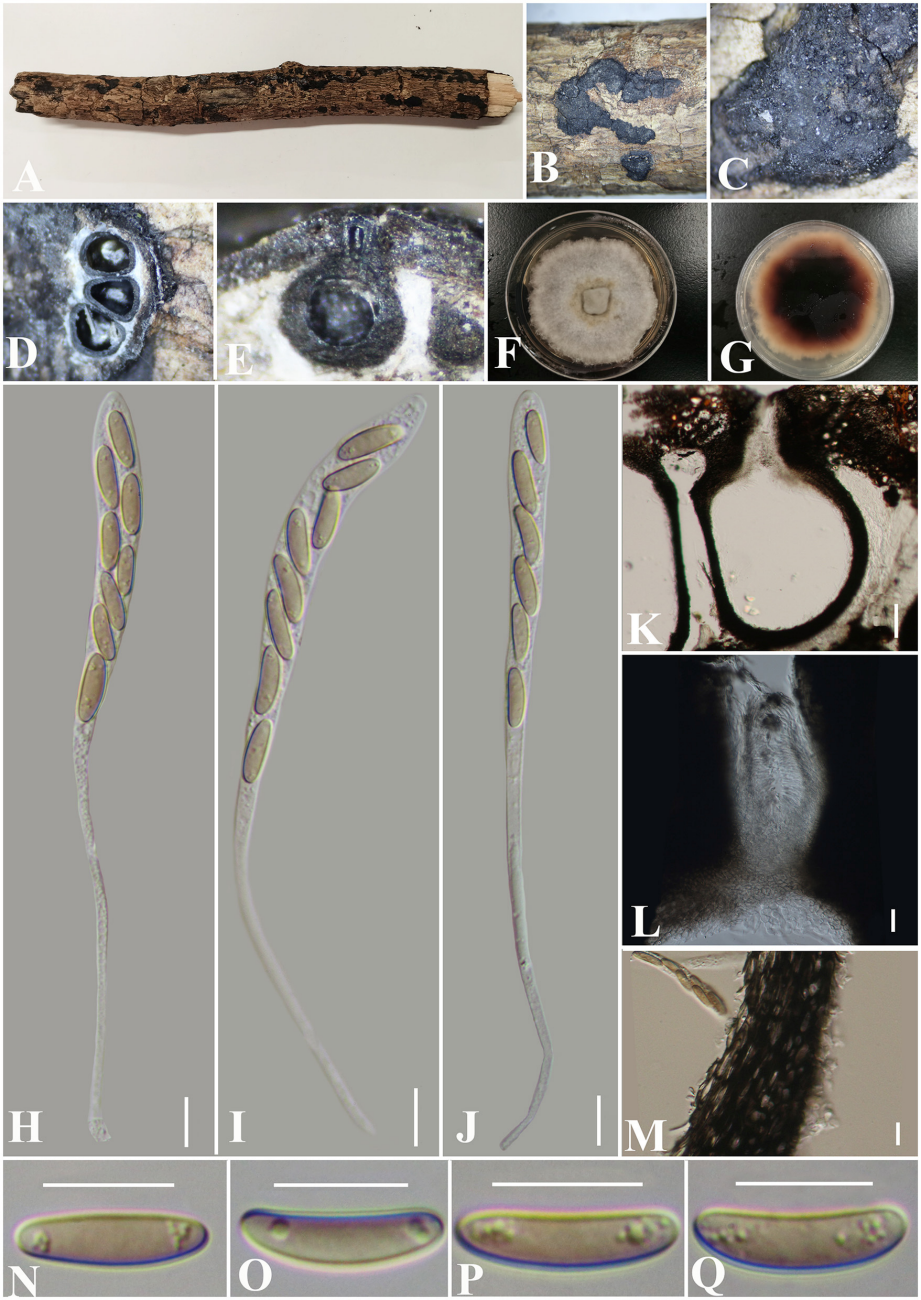
***Diatrypella fatsiae-japonicae*** (GMB0422, holotype). **(A)** Type material. **(B, C)** Close-up of stromata. **(D)** Transverse section through stromata. **(E)** Vertical section through stromata. **(F, G)** Cultures on PDA. **(H–J)** Asci. **(K)** Section through the ascostroma. **(L)** Ostiolar canal. **(M)** Peridium. **(N–Q)** Ascospores. Scale bars: **(H–J, L–Q)** = 10 μm; **(K)** = 50 μm.

***Diatrypella fatsiae-japonica*** S. H. Long & Q. R. Li. sp. nov. (Figure 8).

**MycoBank No**: MB 846767.

**Etymology**: Refers to the host of *Fatsia japonica* (Thunb.) Decne.

**Material examined**: China, Guizhou Province, Qiannan Buyi Miao Autonomous Prefecture, Lan Ding Mountain (25°28′58.27″N, 107°53′53.70″E) on branches of *Fatsia japonica* (Thunb.) Decne. et Planch., 12 June 2021, Altitude: 545 m, S. H. Long & Q. R. Li., LDS61 (GMB0422, **holotype**), ex-type GMBC0422; *ibid* (KUN-HKAS 126460, **isotype**).

*Saprobic* on the surface of dead branches of *Fatsia japonica*. **Sexual morph:** ***Stromata*** 0.4–0.7 cm long and 0.4–0.6 cm broad ($\bar{\text{x}}$ = 0.6 × 0.4 mm, *n* = 30), ~0.6 mm thick, well-developed, erumpent through the bark, irregular in shape, effused, sometimes patch-like, pustulate, rugose, visible as black, solitary to gregarious, numerous ascomata immersed in one stroma. ***Endostroma*** consists of an outer layer of black, small, dense, thin parenchymal cells and an inner layer of white, large, loose parenchymal cells. ***Perithecia*** 285–557.5 μm high, 223.5–320 μm diameter ($\bar{\text{x}}$ = 510.5 × 259.7 μm, *n* = 10), semi-immersed in the stroma, globose to subglobose with a long cylindrical neck in the stroma. ***Ostioles*** opening separately, papillate, central. ***Peridium*** 25–40 μm thick, dark brown to hyaline with *textura angularis* cell layers. ***Asci*** 150.5–186 × 8–10 μm ($\bar{\text{x}}$ = 165.6 × 8.8 μm *n* = 30), 8-spored, unitunicate, long-cylindrical, with long stipe, rounded, apical rings inamyloid. ***Ascospores*** 10–17.5 × 3–4.5 μm ($\bar{\text{x}}$ = 13 × 3.9 μm, *n* = 30), overlapping, ellipsoid to allantoid, straight or slightly curved, light olivaceous, smooth, aseptate, usually with small guttules. **Asexual morph**: Undetermined.

**Culture characteristics**: Ascospores germinating on PDA within 24 h. Colonies on PDA, white when young, became light brown, dense, but thinning toward the edge, margin rough, white from above, reverse white at the margin, mauve to sepia and at the center, no pigmentation, and no sporulation produced on the PDA medium.

**Additional material examined**: China, Guizhou Province, Qiannan Buyi Miao Autonomous Prefecture, Lan Ding Mountain (25°28′31.28″N, 107°53′13.38″E) on branches of an unidentified plant, 12 June 2021, Altitude: 833 m, S. H. Long & Q. R. Li., LDS107 (GMB0423, **paratype**, ex-paratype GMBC0423).

**Notes**: Figure 1 shows that the GMB0422 is located in the unsolved clade which contains *Diatrype* and *Diatrypella*. However, the ascospores of GMB0422 are longer than those of *Diatrypella favacea* (10–17.5 vs. 6–8 μm) (Vasilyeva and Stephenson, 2005). *Diatrypella guiyangensis, Diatrype lancangensis*, and *Diatrype palmicola* have 8-spored asci; however, the ascospores of GMB0422 are wider than those of *Diatrype langcangensis* (10–17.5 × 3–4.5 μm vs. 11–18.5 × 2–4 μm) (Long et al., 2021) and larger than those of *Diatrype palmicola* (10–17.5 × 3–4.5 μm vs. 7–8 × 1.5–2 μm) (Liu et al., 2015), and the ascospores of GMB0422 are light olivaceous which are different from brown to dark brown in *Diatrype lancangensi*s and hyaline in *Diatrypella guiyangensis* (Long et al., 2021).

**Figure 9:**
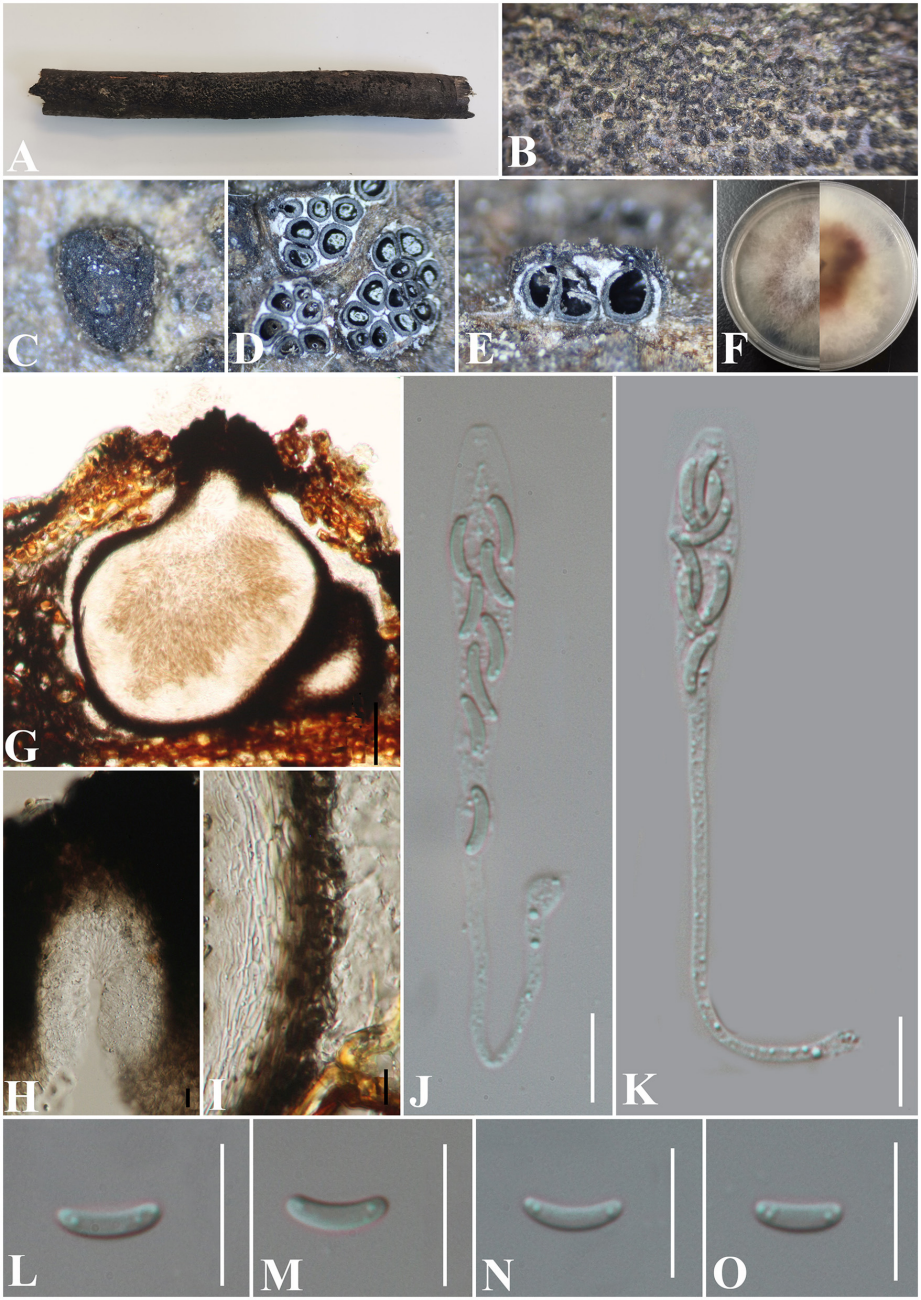
***Diatrypella guiyangensis*** (GMB0414, holotype). **(A)** Type material. **(B, C)** Close-up of stromata. **(D)** Transverse section through stromata. **(E)** Vertical section through stromata. **(F)** Cultures on PDA. **(G)** Section through the ascostroma. **(H)** Ostiolar canal. **(I)** Peridium. **(J, K)** Asci. **(L–O)** Ascospores. Scale bars: **(G)** = 50 μm; **(H–O)** = 10 μm.

***Diatrypella guiyangensis*** S. H. Long & Q. R. Li. sp. nov. (Figure 9).

**MycoBank No**: MB 846766.

**Etymology**: Refers to the collection area of type specimens, Guiyang city.

**Material examined**: China, Guizhou Province, Guiyang City, Guiyang Medical University (26°22′31.28“N, 106°38′18.38″E) on branches of an unidentified plant, 1 August 2020, Altitude: 1128 m, S. H. Long & Q. R. Li., 2020G24 (GMB0414, **holotype**), ex-type GMBC0414; *ibid* (KUN-HKAS 126457, **isotype**).

*Saprobic* on the bark of an unidentified plant branch. **Sexual morph:** ***Stromata*** erumpent through the bark, extending into a black area, postulate to irregular in shape, rugose, gregarious, 3–10 ascomata immersed in one stroma, 0.9–1.3 mm diameter ($\bar{\text{x}}$ = 1.1 × 1.2 mm, *n* = 30), ~0.6 mm high. ***Endostroma*** consists of an outer layer of black, small, dense, thin parenchymal cells and an inner layer of white, large, loose parenchymal cells. ***Perithecia*** 530–640 μm high, 250–425 μm diameter ($\bar{\text{x}}$ = 563.8 × 336.2 μm, *n* = 10), embedded in bark, globose to subglobose with cylindrical neck. ***Ostioles*** opening separately, papillate, central. ***Peridium*** 40–60 μm thick, dark brown to hyaline with *textura angularis* cell layers. ***Asci*** (71) 86.5–126.5 × 4.6–8 μm ($\bar{\text{x}}$ = 99 × 6.7 μm, *n* = 30), 8-spored, unitunicate, long-cylindrical, with long stipe, rounded to truncate apex, apical rings inamyloid. ***Ascospores*** 6.5–8 × 1–2 μm ($\bar{\text{x}}$ = 7.3 × 1.5 μm, *n* = 30), overlapping, allantoid, slightly curved, subhyaline, smooth, aseptate, usually with two small guttules. **Asexual morph**: Undetermined.

**Culture characteristics**: Ascospores germinating on PDA within 24 h. Colonies on PDA, white when young, became mauve, dense, but thinning toward the edge, margin rough, white from above, reverse white at the margin, mauve to luteous at the center, no pigmentation, and no sporulation produced on the PDA medium.

**Additional material examined**: China, Guizhou Province, Guiyang City, Guiyang Medical University (26°22′73.90″N, 106°39′10.88″E) on branches of an unidentified plant, 8 August 2022, Altitude: 1147 m, S. H. Long & Q. R. Li., 2020G53 (GMB0415, **paratype**, ex-paratype GMBC0415).

**Notes:** In Figure 1, GMB0414 was closely related to species of *Diatrype* and *Diatrypella*, but *Diatrypella favacea* has polysporous asci, whereas GMB0414 has only eight ascospores (Croxall, 1950; Glawe and Rogers, 1984). Moreover, the asci of GMB0414 are longer than those of *Diatrypella favacea* (86.5–126.5 × 4.6–8 μm vs. 70–90 × 8–12 μm) (Vasilyeva and Stephenson, 2005). *Diatrypella pulvinata* was introduced as an asexual fungus on a branch of *Quercus garryana* (Zhu et al., 2021). *Diatrype lancangensis* and *Diatrype palmicola* have 8-spored asci, but the stromata of both species are flat, whereas the stromata of GMB0414 are verrucose to conical, and the ascospores of GMB0414 are smaller than those of *Diatrype langcangensis* (6.5–8 × 1–2 μm vs. 11–18.5 × 2–4 μm), and the asci are larger than those of *Diatrype palmicola* (86.5–126.5 × 4.6–8 μm vs. 70–110 × 7–9 μm) (Liu et al., 2015).

***Paraeutypella*** L.S. Dissan., J.C. Kang, Wijayaw. & K.D. Hyde, Biodiversity Data Journal 9: e63864, 11 (2021).

**MycoBank No**: MB 557954.

**Note**: *Paraeutypella* was introduced by Dissanayake et al. (2021) and was typified by *P. guizhouensis* L.S. Dissan., J.C. Kang & K.D. Hyde. The genus shows eutypella-like morphology (Dissanayake et al., 2021), having immersed stromata with elongated ostiolar neck, 8-spored, clavate to cylindrical clavate or spindle-shaped asci, allantoid ascospores. The asexual morph was reported as coelomycetous (Vasilyeva and Stephenson, 2006). In this study, we introduce a new species of *Paraeutypella* from China.

**Figure 10:**
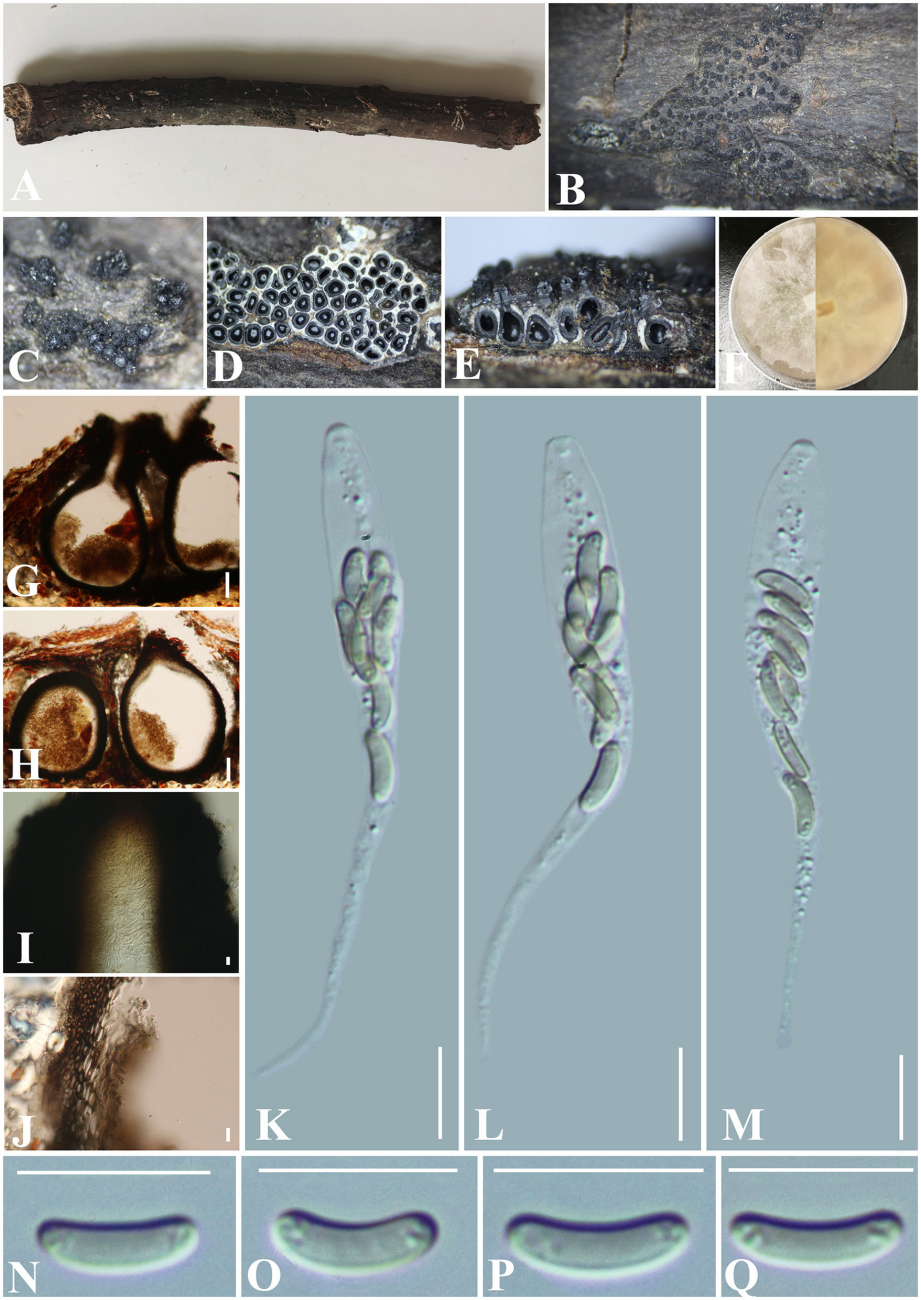
***Paraeutypella subguizhouensis*** (GMB0420, holotype). **(A)** Type material. **(B, C)** Close-up of stromata. **(D)** Transverse section trough stromata. **(E)** Vertical section through stromata. **(F)** Cultures on PDA. **(G, H)** Sections through the stromata. **(I)** Ostiolar canal. **(J)** Peridium. **(K–M)** Asci. **(N–Q)** Ascospores. Scale bars: **(G, H)** = 100 μm; **(I–Q)** = 10 μm.

***Paraeutypella subguizhouensis*** S. H. Long & Q. R. Li. sp. nov. (Figure 10).

**MycoBank No**: MB 846772.

**Etymology**: Morphologically similar to *Paraetypella guizhouensis*.

**Material examined**: China, Guizhou Province, Guiyang City, Guiyang Forest Park (26°32′52.79″N, 106°45′10.31″E) on branches of an unidentified plant, 22 June 2021, Altitude: 1165 m, S. H. Long & Q. R. Li., GYSLGY22 (GMB0420, **holotype**), ex-type GMBC0420; *ibid* (KUN-HKAS 126462, **isotype**).

*Saprobic* on the surface of dead wood. **Sexual morph:** ***Stromata*** poorly developed, immersed in bark, aggregated, circular to irregular in shape, 0.4–1.5 cm long and 0.3–1 cm broad ($\bar{\text{x}}$ = 0.9 × 0.5 cm, *n* = 30), ~1 mm thick, numerous ascomata immersed in one stroma showing clustered beaks. ***Endostroma*** consists of an outer layer of black, small, dense, thin parenchymal cells and an inner layer of white, large, loose parenchymal cells. ***Perithecia*** 720–860 μm high, 280–335 μm diameter ($\bar{\text{x}}$ = 807.2 × 308.3 μm, *n* = 10), semi-immersed in the stroma, globose to subglobose with a long cylindrical neck (350–410 μm) in and out of the bark. ***Ostioles*** opening separately from the top of the neck, papillate, central. ***Peridium*** 50–80 μm thick, dark brown to hyaline with *textura angularis* cell layers. ***Asci*** 61–90 × 6–7.5 μm ($\bar{\text{x}}$ = 79 × 7.2 μm *n* = 30), 8-spored, unitunicate, long-cylindrical, with long stipe, rounded to truncate apex, apical rings inamyloid. ***Ascospores*** 7.5–10.5 × 1.5–2.5 μm ($\bar{\text{x}}$ = 8.8 × 2.2 μm, *n* = 30), overlapping, allantoid, slightly curved, subhyaline, smooth, aseptate, usually with small guttules. **Asexual morph**: Undetermined.

**Culture characteristics**: Ascospores germinating on PDA within 24 h. Colonies on PDA, white when young, became mauve, dense but thinning toward the edge, margin rough, white from above, reverse mauve to luteous, no pigmentation, and no sporulation produced on the PDA medium.

**Additional material examined:** China, Guizhou Province, Guiyang City, Guiyang Forest Park (26°32′77.35”N, 106°44′19.92″E) on branches of an unidentified plant, 23 June 2022, Altitude: 1165 m, S. H. Long & Q. R. Li., GYSLGY51 (GMB0421, **paratype**, ex-paratype GMBC0421).

**Notes:** In stromatal morphology, *Paraeutypella subguizhouensis* (GMB0420) resembles the species of *Paraeutypella* (Dissanayake et al., 2021). In our phylogenetic analyses, GMB0420 was accommodated in *Paraeutypella s. str*. (Figure 1). *Paraeutypella psedoguizhouensis* can differ from other species of *Paraeutypella* in having more than 25 ascomata in one stroma, however, species of *Prareutypella* only have 4–25 ascomata immersed in one stroma (Dissanayake et al., 2021). Moreover, GMB0420 differs from *Paraetypella guizhouensis* in having a shorter ostiolar neck (350–410 μm vs. 400–418 μm) (Dissanayake et al., 2021), from *Paraetypella vitis* in having longer asci (61–90 × 6–7.5 μm vs. 40–46 × 6–8 μm) and smaller ascospores (7.5–10.5 × 1.5–2.5 μm vs. 9.6–12 × 2–2.4 μm) (Glawe and Jacobs, 1987), and from *Paraeytypella citricola* by having smaller ascospores (7.5–10.5 × 1.5–2.5 μm vs. 10–12 × 2–3 μm) (Trouillas et al., 2011). The phylogenetic position of *Allocryptovalsa castaneicola* is consistent with the previous article (Zhu et al., 2021), and it was introduced as a species of *Allocryptovalsa* since it has polyspored asci. *Paraeutypella subguizhouensis* differs from *Allocryptovalsa castaneicola* in having shorter ascospores (7.5–10.5 × 1.5–2.5 μm vs. 22–25 × 5–6 μm) (Zhu et al., 2021). Here, we temporarily classify it as *Paraeutypella* until the classification of *Diatrypaceae* is clearer at the genus level.

***Peroneutypa*** Berl., Icon. fung. (Abellini) 3(3-4): 80 (1902).

**MycoBank No**: MB 3834.

**Notes**: *Peroneutypa* was introduced by Berlese (1902) for having valsoid stroma with long prominent necks, sessile to long stalks, small, clavate asci with truncated apices, and allantoid ascospores (Saccardo and Saccardo, 1905; Carmarán et al., 2006, 2014). Rappaz (1987) proposed *P. bellula* (Desm.) Berl. as the type species of *Peroneutypa*. The asexual morph of this genus is not reported so far.

**Figure 11:**
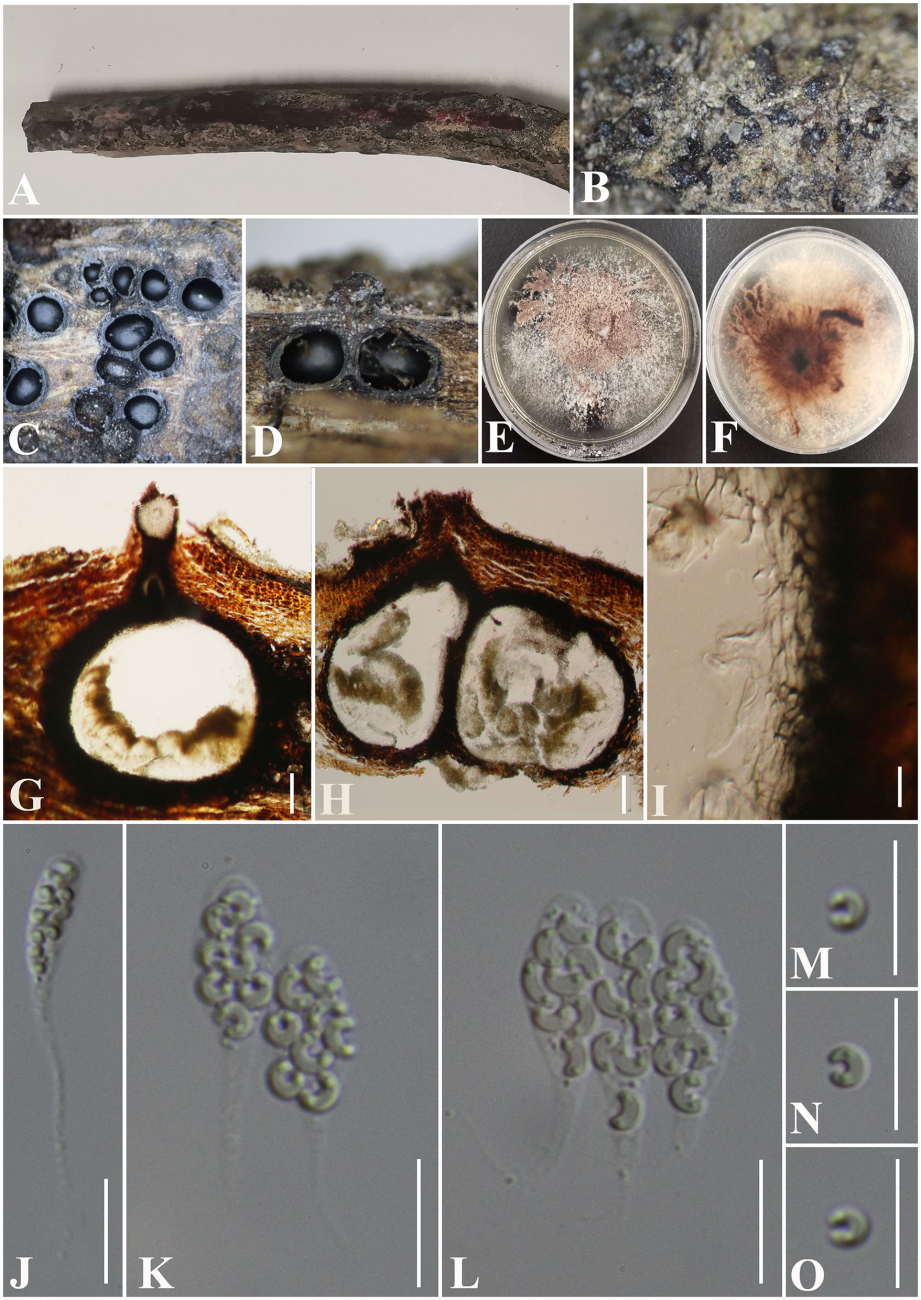
***Peroeutypa hainanensis*** (GMB0424, holotype). **(A)** Type material. **(B)**. Close-up of stromata. **(C)** Transverse section through stromata. **(D)** Vertical section through stromata. **(E, F)** Culture on PDA. **(G, H)** Sections through the ascostroma. **(I)** Peridium. **(J–L)** Asci. **(M–O)** Ascospores Bar: **(G, H)** =100 μm; **(I–O)** = 10 μm.

***Peroeutypa hainanensis*** S. H. Long & Q. R. Li. sp. nov. (Figure 11).

**MycoBank** No: MB 846770.

**Etymology**: Refers to the collection area, Hainan Province.

**Material examined**: China, Hainan Province, Wenchang City, Tongguling Nature Reserve (19°39'16.23“N, 111°1'38.68”E) on branches of an unidentified plant, 12 November 2021. Altitude: 67 m, S. H. Long & Q. R. Li., TGL4 (GMB0424, **holotype**), ex-type GMBC0424; *ibid* (KUN-HKAS 126463, **isotype**).

*Saprobic* on dead branches of an unidentified plant. **Sexual morph**: ***Stromata*** 0.4–0.7 mm diameter × 0.1–0.3 mm long, non-sulcate, poorly developed, solitary to gregarious, immersed, ostiolar canals raised to erumpent the surface of stromata, dark brown to black, 1–7 perithecia immersed in one stroma. ***Perithecia*** 350–600 μm high × 130–300 μm diameter ($\bar{\text{x}}$ = 375 × 202 μm, *n* = 10), immersed, globose to subglobose, brown to black, ostiolate. ***Ostiolar canal*** 105–420 μm high, 80–120 μm diameter ($\bar{\text{x}}$ = 265 × 100 μm, *n* = 25), cylindrical, sulcate, at the apex curved, periphysate. ***Peridium*** 45–65 μm wide, composed of two layers, outer section dark brown to black, thick-walled cells, arranged in textura globulosa to *textura angularis*, inner part comprising hyaline textura angularis cells. ***Asci*** 28.5–40 × 3.5–6.5 μm ($\bar{\text{x}}$ = 33.5 × 5.5 μm, *n* = 30), 8-spored, unitunicate, clavate, with long stipitate, apically rounded to truncate, apical rings inamyloid. ***Ascospores*** 5.0–7.3 × 1–2 μm ($\bar{\text{x}}$ = 6 × 1.5 μm *n* = 30), overlapping, allantoid, strongly curved, subhyaline, with small guttules at ends. **Asexual morph**: Undetermined.

**Culture characteristics**: Ascospores germinating on PDA after 24 h. Colonies white when young, became pale brown circular to irregular, medium dense, flat or effuse, slightly raised, fluffy to powder, margin rough, white at the margin and light brown at the center from below, no pigmentation, and no sporulation produced on the PDA medium.

**Additional material examined**: China, Hainan Province, Wenchang City, Tongguling Nature Reserve (19°39′38.81″N, 111°0′50.82″E) on branches of an unidentified plant, 12 November 2021. Altitude: 73 m, S. H. Long & Q. R. Li., TGL53 (GMB0425, **paratype**, ex-paratype GMBC0425).

**Notes**: Figure 1 shows that *Peroeutypa hainanensis* clustered with species of *Peroneutypa*. Morphologically, the ascospores of *P. obesa* and *P. curvispora* are strongly curved, but the ascospores of *Peroeutypa hainanensis* (5.0–7.3 × 1–2 μm) are longer than those of *P. curvispora* (3.0–4.5 × 1–1.5 μm) (Carmarán et al., 2006; Shang et al., 2018). The spiny or bristly appearance of the stromata surface of *P. obesa* can be distinguished from *P. hainanensis*, and the stromata of GMB0424 are smaller than those of *P. obesa* (10–15 mm diameter × 7–10 m long) (Rappaz, 1987). Based on both molecular and phylogenetic analyses, here, we introduce new species, *Peroeutypa hainanensis*.

**Figure 12:**
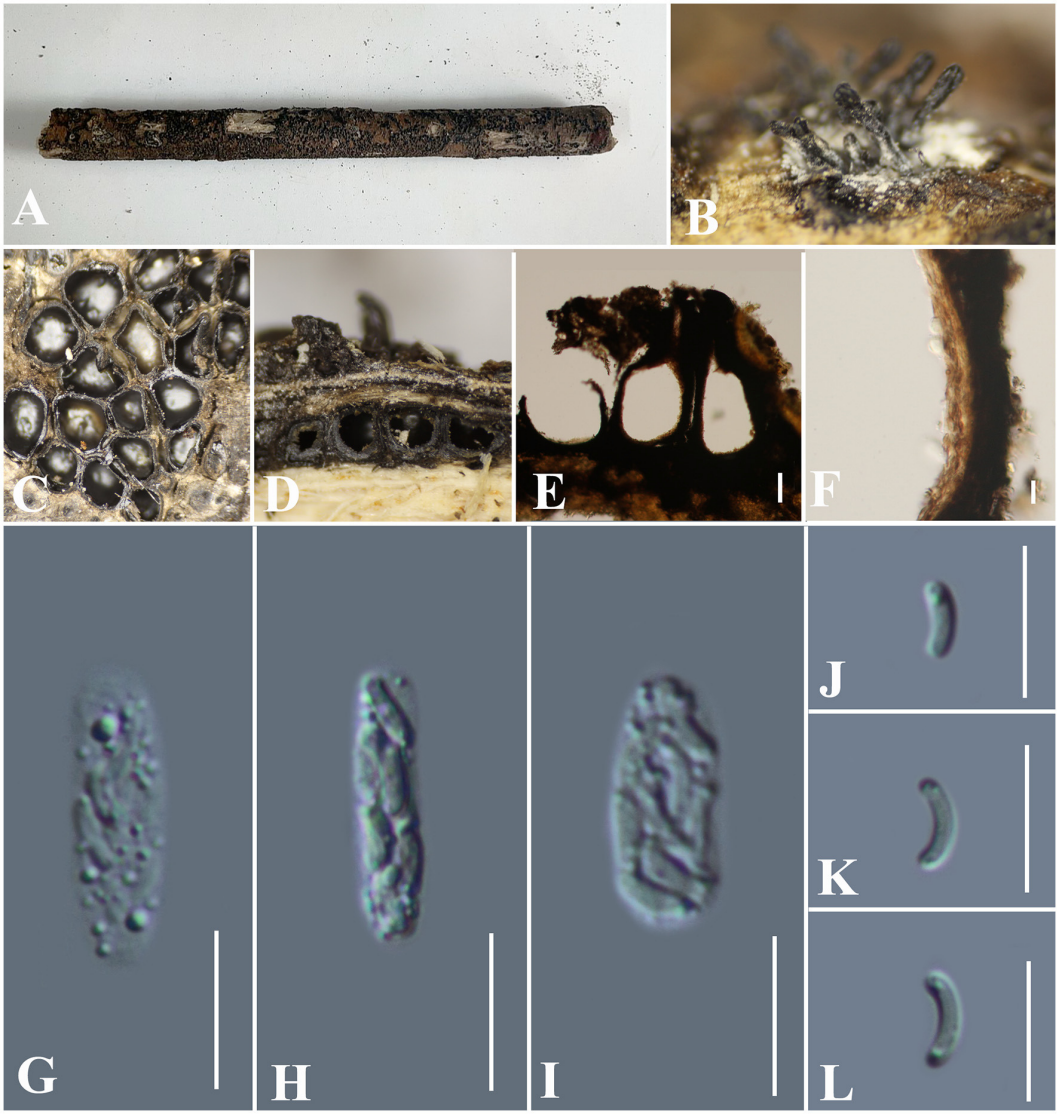
***Peroneutypa qianensis*** (GMB0431, holotype). **(A)** Type material. **(B)** Close-up of stromata. **(C)** Transverse section through stromata. **(D)** Vertical section through stromata. **(E)** Section through the ascostroma. **(F)** Peridium. **(G–I)** Asci. **(J–L)** Ascospores. Scale bars: **(E)** = 100 μm; **(F–L)** = 10 μm.

***Peroneutypa qianensis*** S. H. Long & Q. R. Li. sp. nov. (Figure 12).

**MycoBank** No: MB846771.

**Etymology**: Refers to the location of the type specimen, Qian is the abbreviation of Guizhou Province in Chinese.

**Material examined**: China, Guizhou Province, Qiannan Buyi Miao Autonomous Prefecture, Maolan National Nature Reserve (25°18′2.76″N, 108°4′29.48″E) on branches of an unidentified plant, 7 July 2021. Altitude: 545 m, S. H. Long & Q. R. Li., MLB62 (GMB0431, **holotype**), ex-type GMBC0431; *ibid* (KUN-HKAS 126464, **isotype**).

*Saprobic* on dead branches of an unidentified plant. **Sexual morph**: ***Stromata*** poorly developed, 1.5–2 mm wide, solitary to gregarious, immersed; ostiolar canals raised to erumpent the surface of stromata, dark brown to black, non-sulcate. ***Perithecia*** 320–540 μm high × 175–290 μm diameter ($\bar{\text{x}}$ = 375 × 202 μm, *n* = 10), immersed, globose to subglobose, brown to black, ostiolate. ***Ostiolar canal*** 105–420 μm high, 80–120 μm diameter ($\bar{\text{x}}$ = 265 × 100 μm, *n* = 25), cylindrical, sulcate, at the apex curved, periphysate. ***Peridium*** 45–65 μm wide, composed of two layers, outer section dark brown to black, thick-walled cells, arranged in *textura* globulosa to *textura angularis*, inner part comprising hyaline textura angularis cells. ***Asci*** 16.5–20.5 × 4–6 μm ($\bar{\text{x}}$ = 18.4 × 5 μm, *n* = 30), 8-spored, unitunicate, clavate, sessile, apically rounded to truncate, apical rings inamyloid. ***Ascospores*** 4.5–6.3 × 1.5–0.3 μm ($\bar{\text{x}}$ = 5.6 × 1.8 μm *n* = 30), overlapping, allantoid, straight to slightly curved, subhyaline, with small guttules at ends. **Asexual morph**: Undetermined.

**Culture characteristics**: Ascospores germinating on PDA after 24 h. Colonies white when young, became pale brown circular to irregular, medium dense, flat or effuse, slightly raised, fluffy to powder, margin rough, white at the margin and light brown at the center from below, no pigmentation, and no sporulation produced on the PDA medium.

**Additional material examined**: China, Guizhou Province, Qiannan Buyi Miao Autonomous Prefecture, Maolan National Nature Reserve (25°17′52.14″N, 108°4′27.01″E) on branches of an unidentified plant, 7 July 2021. Altitude: 651 m, MLB150 (GMB0432, **paratype**, ex-paratype, GMBC0432).

**Note:** Our phylogenetic analyses (Figure 1) show that *Peroneutypa qianensis* resides as the sister clade to *P. mackenziei*, with high bootstrap and PP values (99/1). Morphologically, *P. qianensis* is similar to *P. mackenziei* (MFLU 16-1441, holotype), in that both of them have the clavate, sessile ascospores (Shang et al., 2017). However, the ascospores of *P. mackenziei* are narrower than those of the new specimen GMB0431 (4.5–6.5 × 1–2 μm vs. 4.5–6.3 × 1.5–3 μm) (Shang et al., 2017). Combining morphological and molecular data, we introduce GMB0431 as a new species of *Peroneutypa*.

***Vasilyeva*** S. H. Long, Wijayaw. & Q. R. Li. gen. nov.

**MycoBank No**: MB846773.

**Etymology**: We dedicate this genus to L.N. Vasilyeva, an excellent taxonomist who extensively worked on *Diatrypacaea* research in China.

*Saprobic* on an unidentified wood. **Sexual morph**: ***Stromata*** poorly developed, immersed in the host tissue, showing a long beak higher than the wood surface and a long channel immersed, the beak in the air covered with the long setae. ***Perithecia*** with a long beak, scattered or in rows, circular to oblate. ***Ostioles*** apparent on the surface of the substrate, higher than the surface of the wood, emerging on the surface separately. ***Asci*** 8-spored, unitunicate, clavate to long-cylindrical, with long stipe, apically rounded, apical rings inamyloid. ***Ascospores*** overlapping, allantoid, straight or slightly curved, subhyaline to hyaline, with oil droplets at ends. **Asexual morph**: Undetermined.

Type species: ***Vasilyeva cinnamomi*** S. H. Long, Wijayaw. & Q. R. Li.

**Notes:** The genus *Vasilyeva* is introduced to accommodate the new collection made from Hainan, China. Figure 1 shows that the new collection formed a distinct branch which is sister to *Peroneutypa*. Morphologically, *Vasilyeva* has stromata covered with long setae, long stipe asci, allantoid, and straight or slightly curved ascospores. The perithecia of *Vasilyeva cinnamomi* are immersed in the stromata with a long beak which includes a part higher than the surface of wood covered with long setae and a long channel immersed, and the ostioles emerging on the surface separately. It is different from all genera in *Diatrypaceae*. Based on morphological and phylogenetic analyses, *Vasilyeva* was proposed as a new genus.

**Figure 13:**
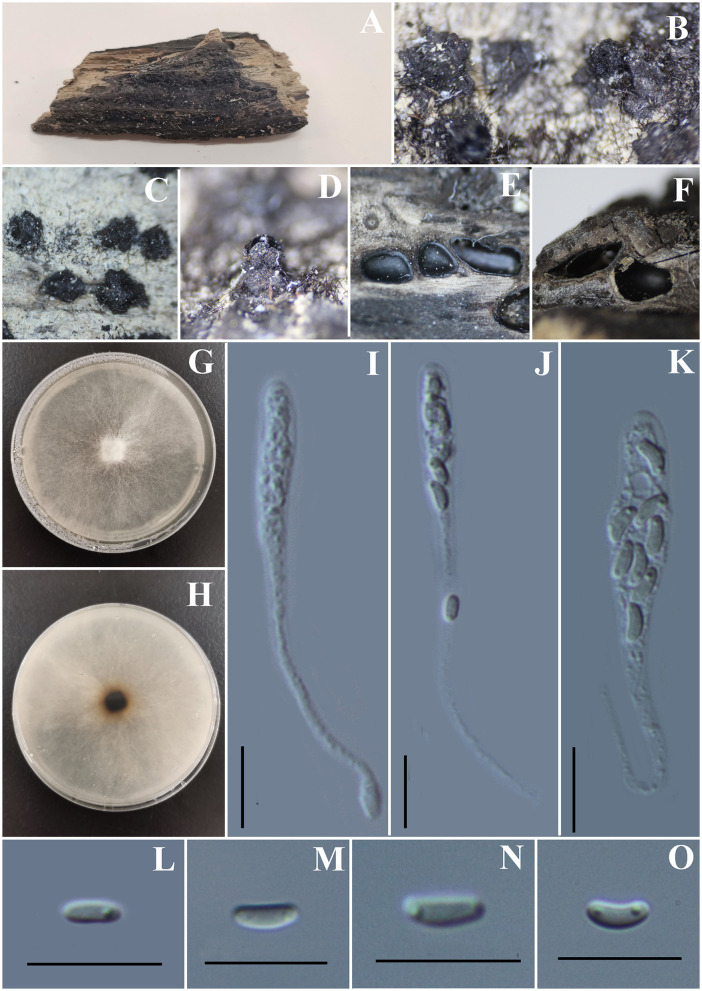
***Vasilyeva cinnamomi*** (GMB0418, holotype). **(A)** Type material. **(B–D)** Close-up of stromata. **(E)** Transverse section through stromata. **(F)** Vertical section through stromata. **(G, H)** Culture on PDA. **(I–K)** Asci. **(L–O)** Ascospores. Scale bars: **(C–F)** = 1 mm; **(I–O)** = 10 μm.

***Vasilyeva cinnamomi*** S. H. Long, Wijayaw. & Q. R. Li sp. nov. (Figure 13).

**MycoBank No**: 846774.

**Etymology**: Refers to its host, *Cinnamomum cinnamomi* (L.) Presl.

**Material examined**: China, Hainan Province, Wuzhishan City Wuzhishan Nature Reserve (18°54'21.47“N, 109°40'57.99”E) on wood chips of *Cinnamomum cinnamomi* (L.) Presl, 15 November 2021. Altitude: 795 m, S. H. Long & Q. R. Li., WZS28 (GMB0418, **holotype**), ex-type GMBC0418; *ibid* (KUN-HKAS 126465, **isotype**).

*Saprobic* on dead wood chips of *Cinnamomum cinnamomi*. **Sexual morph**: ***Stromata*** poorly developed, immersed in the host tissue, showing a black beak on the wood surface. ***Perithecia*** 0.8–1.3 mm high × 1.3 – 2.3 mm diameter ($\bar{\text{x}}$ = 0.9 × 1.8 mm, *n* = 10) (the length of the beak is not included), with a long beak [partly in the wood (0.6–0.8 mm high) and partly on the surface of the wood (0.5–0.9 mm) covered with the long setae], scattered or in rows, circular to oblate. ***Ostioles*** apparent on the surface of the substrate, higher than the surface of wood, emerging on the surface separately. ***Asci*** 58 – 77.5 × 4 – 7 μm ($\bar{\text{x}}$ = 66.7 × 5.1 μm, *n* = 30), 8-spored, unitunicate, clavate to long-cylindrical, with long stipe, apically rounded, apical rings inamyloid. ***Ascospores*** 4.0 – 6.0 × 1.5 – 2.5 μm ($\bar{\text{x}}$ = 4.7 × 1.9 μm *n* = 30), overlapping, allantoid, straight or slightly curved, subhyaline to hyaline, with small guttules at ends. **Asexual morph**: Undetermined.

**Culture characteristics:** Ascospores germinating on PDA within 24 h. Colonies on PDA, white when young, became brown, dense, but thinning toward the edge, margin rough, white from above, reverse white to brown, no pigmentation, and no sporulation produced on the PDA medium.

**Additional Material examined**: China, Hainan Province, Wuzhishan City Wuzhishan Nature Reserve (18°54′70.43″N, 109°41′10.59″E) on branches of an unidentified plant, 15 November 2021. Altitude: 833 m, S. H. Long & Q. R. Li., WZS90 (GMB0419, **paratype**, ex-paratype GMBC0419).

**Notes:** *Vasilyeva cinnamomi* is a morphologically and phylogenetically distinct species from other known species in *Diatrypaceae*. A peculiar feature of *Vasilyeva cinnamomi* is the ostioles appearing separately on the surface and the perithecia which are immersed in the stromata with a long beak are higher than the surface of the wood.

## Discussion

*Diatrypaceae* species have a cosmopolitan distribution and often inhabit the deadwood and bark of many plant species. However, the generic concepts of *Diatrypaceae* have been unstable; thus, many species were transferred from one genus to another (Phookamsak et al., 2019; Konta et al., 2020).

In this study, one new genus and eight new species were described based on phylogenetic analyses and morphological characteristics. The new genus *Vasilyeva* differs from other genera in its perithecia which have two parts, the lower part is immersed in the stromata, and the higher part has a long beak and is higher than the surface of the wood. *Diatrype camelliae-japonicae, Diatrype rubi, Diatrypella guiyangensis, Diatrypella fatsiae-japonicae, Peroneutypa hainanensis, Peroneutypa qianensis*, and *Paraeutypella subguizhouensis* have been introduced as novel taxa from various substrates in Guizhou and Hainan provinces, China.

In addition, *Allocryptovalsa rabenhorstii* and *Diatrype enteroxantha* have been reported from China for the first time. Two known species of *Allocryptovalsa xishuangbanica* and *Diatrype betulae* were described and illustrated, of which *Allocryptovalsa xishuangbanica* was the first reported from Guizhou province from China. Based on the phylogenetic analyses and megablast, we conclude GMB0426 is representing the sexual morph of *Diatrype betulae*, and this is the first time reporting its sexual morph.

Our phylogenetic analyses show that the division of genera is confusing which is consistent with the previous studies (Acero et al., 2004; Trouillas et al., 2011; Mehrabi et al., 2015, 2016; de Almeida et al., 2016; Shang et al., 2017; Dissanayake et al., 2021; Long et al., 2021; Zhu et al., 2021; Ma et al., 2023). Compared to the number of *Diatrypaceae* species, the available sequences in NCBI are relatively fewer. Most species in *Diatrypaceae* are lacking DNA sequences. Moreover, several genera (e.g., *Dothideovalsa, Echinomyces, Endoxylina*, and *Rostronitschkia*) still have no available sequences. The current molecular phylogenetic study of *Diatrypaceae* only uses ITS and β-tubulin gene sequences, which do not distinguish this family well, and we believe that the sequences of the large subunit (LSU) ribosomal RNA gene and RNA polymerase II second largest subunit (RPB2) gene sequences should be added in future studies for a more accurate phylogenetic analysis of this family.

In our investigation, we found that the molecular data did not correlate well with morphological characteristics, and two materials with 99% similarity differed significantly in morphology. The morphological comparison shows that there is little morphological difference between genera, and the traditional morphological characteristics such as the number of ascospores per ascus and the morphology of stromata do not distinguish well among genera. Long et al. (2021) stated that there are eight ascospores or polysporous in each ascus in different species of the same genus. The number of ascospores in an ascus can no longer be regarded as the main feature of the genus of *Diatrypaceae*, although this feature has been widely used in the establishment of the genus (Glawe and Rogers, 1984; Vasilyeva and Stephenson, 2005; Konta et al., 2020). Vasilyeva (1986) proposed that the morphology of stromata was influenced by the host, environments, and some other factors, and there were limitations in the use of substratum morphology as a basis for the identification which is consistent with our research. The stromata of *Neoeutypella, Allodiatrype, Diatrype, Diatrypella, Allocryptovalsa, Cryptovalsa, Eutypella*, and *Paraeutypella* is similar. Therefore, we consider that the morphological characteristics of the stromata may not be used as a basis for the identification of *Diatrypaceae*. The authority of the number of ascospores in the ascus as the important feature of identification at the species level is also challenging to some extent. These morphological taxonomic features, which were considered to be very important in the early stage, constitute the main taxonomic basis of the current genera of *Diatrypaceae* (Tiffany and Gilman, 1965; Glawe and Rogers, 1984; Rappaz, 1987; Vasilyeva and Stephenson, 2005; Senanayake et al., 2015; Senwanna et al., 2017). However, more and more molecular data show that the classification of these genera is unresolved and inconsistent with their morphology (Konta et al., 2020; Long et al., 2021; Zhu et al., 2021; Ma et al., 2023). We must admit that the main DNA sequences currently used for the systematics of *Diatrypaceae* only include ITS and BT, which is not so sufficient. Does the systematics of *Diatrypaceae* need to be started from scratch? We do not have a clear answer yet. However, we believe that *Diatrypaceae* needs to be revised at the genus level, based on type materials, newly collected specimens, more DNA sequences, more suitable morphological features, and other features in the future.

## Data availability statement

The datasets presented in this study can be found in online repositories. The names of the repository/repositories and accession number(s) can be found in the article/supplementary material.

## Author contributions

Y-QK and XZ conceived and designed the experiments. H-MH, Y-PW, Q-ZW, and YL performed the experiment. Q-RL and S-HL analyzed the data and wrote the manuscript. NW, J-CK, and JK provided some materials and polished the language. Y-QK and X-CS revised and approved the final version of the manuscript. All authors contributed extensively to the study presented in the manuscript.
